# Combined Machine Learning and Molecular Modelling Workflow for the Recognition of Potentially Novel Fungicides

**DOI:** 10.3390/molecules25092198

**Published:** 2020-05-08

**Authors:** Ozren Jović, Tomislav Šmuc

**Affiliations:** Ruđer Bošković Institute, Bijenička cesta 54, 10 000 Zagreb, Croatia; Tomislav.Smuc@irb.hr

**Keywords:** classification, regression, docking, drug repurposing, QM/MM, Fe-N(R)C angle

## Abstract

Novel machine learning and molecular modelling filtering procedures for drug repurposing have been carried out for the recognition of the novel fungicide targets of Cyp51 and Erg2. Classification and regression approaches on molecular descriptors have been performed using stepwise multilinear regression (FS-MLR), uninformative-variable elimination partial-least square regression, and a non-linear method called Forward Stepwise Limited Correlation Random Forest (FS-LM-RF). Altogether, 112 prediction models from two different approaches have been built for the descriptor recognition of fungicide hit compounds. Aiming at the fungal targets of sterol biosynthesis in membranes, antifungal hit compounds have been selected for docking experiments from the Drugbank database using the Autodock4 molecular docking program. The results were verified by Gold Protein-Ligand Docking Software. The best-docked conformation, for each high-scored ligand considered, was submitted to quantum mechanics/molecular mechanics (QM/MM) gradient optimization with final single point calculations taking into account both the basis set superposition error and thermal corrections (with frequency calculations). Finally, seven Drugbank lead compounds were selected based on their high QM/MM scores for the Cyp51 target, and three were selected for the Erg2 target. These lead compounds could be recommended for further in vitro studies.

## 1. Introduction

The investigations of novel antifungal compounds and compounds having a synergistic effect on antifungals have been increasing in recent decades [1,2,3,4]. Approximately 215 fungicides have been sorted based on their mode of action (MOA) in the biochemical pathways of plant fungal pathogens in the Fungicide Resistance Action Committee (FRAC) MOA Code List 2019 [1]. For antifungal pesticides, the FRAC grouping is made according to metabolic processes such as respiration and sterol biosynthesis in membranes, with an appropriate intrinsic resistance risk assessment for each corresponding fungicide group [1]. So far, 377 fungicides have been approved for use [2], and only some of them have already been classified according to their MOA.

The article of Alejandro Speck-Planche et al. [3] regards the correct classification of fungicides and inactive compounds while taking into account the different resistance risk categories of the corresponding fungicides. To discriminate between fungicides and non-fungicides besides using a fungicide training set, the authors defined and used a set of inactive compounds or non-fungicides [3]. However, since then (year 2011) some compounds previously declared to be non-antifungals have been studied and occasionally possess some antifungal activities. For example, olanzapine was designated as non-fungicide [3] but was determined later and very recently to be an anti-Cryptococcus drug [5]. Another example is verapamil [3], which later had an inhibitory effect on the *Candida albicans* species [6]. Some other compounds such as Rifampin and Nifedipine, possess a synergistic antifungal effect when combined with some already-established anti-fungal agents [7,8]. Among the 158 used non-fungicides in [3], 27 compounds have been found to possess or might possess some anti-fungal properties (Supplementary Table S1). This might open the door to the question as to what it means to have “a set of non-fungicide compounds”. What is certain is that more and more inactive compounds have been revealed as active compounds toward different species of yeast and/or at least possess a synergistic antifungal effect when combined with already-established fungicides through drug repurposing. Another study of Alejandro Speck-Planche et al. [9] concerns the first multi-species cheminformatics approach for the classification of agricultural fungicide into toxic or nontoxic. That study regards the successful simultaneous assessment of multiple ecotoxicological profiles of agrochemical fungicides or pairs of fungicide-indicator species, of which 81 were fungicides and 20 indicator species [9]. Due to many compounds that have been repurposed very recently as antifungals, in our opinion what is still lacking in the literature is a Drugbank-scaled in silico repurposing study concerning the recognition of novel antifungal agents. This study should establish models based on fungicides′ substructural descriptors that both classifies fungicides into modes of action and also uses these classification models for extrapolation to a large compound data set such as the Drugbank database. This approach still has not been carried out yet to the best of our knowledge.

In other words, this research, using machine learning, is primarily focused on the strategy of identifying (i.e., recognizing) already-known chemical compounds as potential novel antifungal agents that haven′t yet been recognized as such. To do so, in the first part (1) of the study, Drugbank database will be filtered and only compounds specifically similar to fungicides will be further considered as potential hit compounds; while in the second part (2) of the research, all these preselected hit compounds from the Drugbank database will be submitted to extensive docking studies. As a final filtering and confirmation step, we will select only those hits that obtain high enough scores in docking simulations with very specific protein targets.

In this drug repurposing study, we limit our research on finding novel fungicides to a specific fungicide group called inhibitors of sterol biosynthesis, which is the most abundant MOA group “G”—sterol biosynthesis in membranes [1,10]. The most common target protein of that MOA group is known as lanosterol 14-alpha demethylase Cyp51, and the second most important is Erg2 [1,10]. An antifungal compound binds to a specific protein and prevents sterol biosynthesis, which leads to fungal death. Some of the known inhibitors of Cyp51, the target which catalyzes the demethylation of lanosterol to ergosterol, are fluconazole, ketoconazole, simeconazole, and bromuconazole; but the strongest inhibitors reported to date are posaconazole and oteseconazole [11]. Specific chemical functional groups attributed to this G MOA are mostly triazoles and imidazoles, but there are also tetrazoles, pyrimidines, pyridines, and piperazines for Cyp51 inhibitors [10], and morpholines, piperidines, and spiroketalamines for sterol 8,7-isomerase inhibitors [10]. Regarding sterol 8,7-isomerase inhibitors, the already-established fungicides are: aldimorph, dodemorph, fenpropimorph, fenpropidin, piperalin, spiroxamine, and tridemorph [10]. However, regarding Cyp51 inhibitors, there are 36 fungicides in the FRAC code list [10], plus some other fungicides mostly in the triazole or imidazole functional groups [11]. Taking into account some additional fungicides with known (or at least likely) MOAs, an MOA fungicide set which contains 245 compounds is established in this work as an MOA working set (in the following text “MOAW set”; see MOAW set in Supplementary Table S2). In this research, we rely on such a MOAW set because it contains as much sterol biosynthesis inhibitors as possible and also covers quantitatively enough fungicides classified into different fungicide class groups, although there might be big differences in their activities [1]. The possible objection that the FRAC code list deals only with plant antifungals is not a hurdle in this study, because we are not trying to expend the FRAC code list itself, and there are no antifungals from the other FRAC groups reported to date to inhibit either Cyp51 or Erg2 (except the point that group “K” is generally considered to be fungicides with “multi-site activity”, but contains different chemical functional groups than group G). In addition, some FRAC fungicides (e.g., prothioconazole) are already reported to bind to “non-plant” pathogens such as *Candida albicans* [12]. The actual goal is to search for the new fungal protein target inhibitors while repurposing the Drugbank compound set; and FRAC code lists essentially serves as the initial point for creating and testing a discriminant model for finding new hit compounds of the Cyp51 target and Erg2 protein target when using molecular descriptors in the prefiltering procedure, i.e., the first part (1) of the study.

**Scheme 1:**
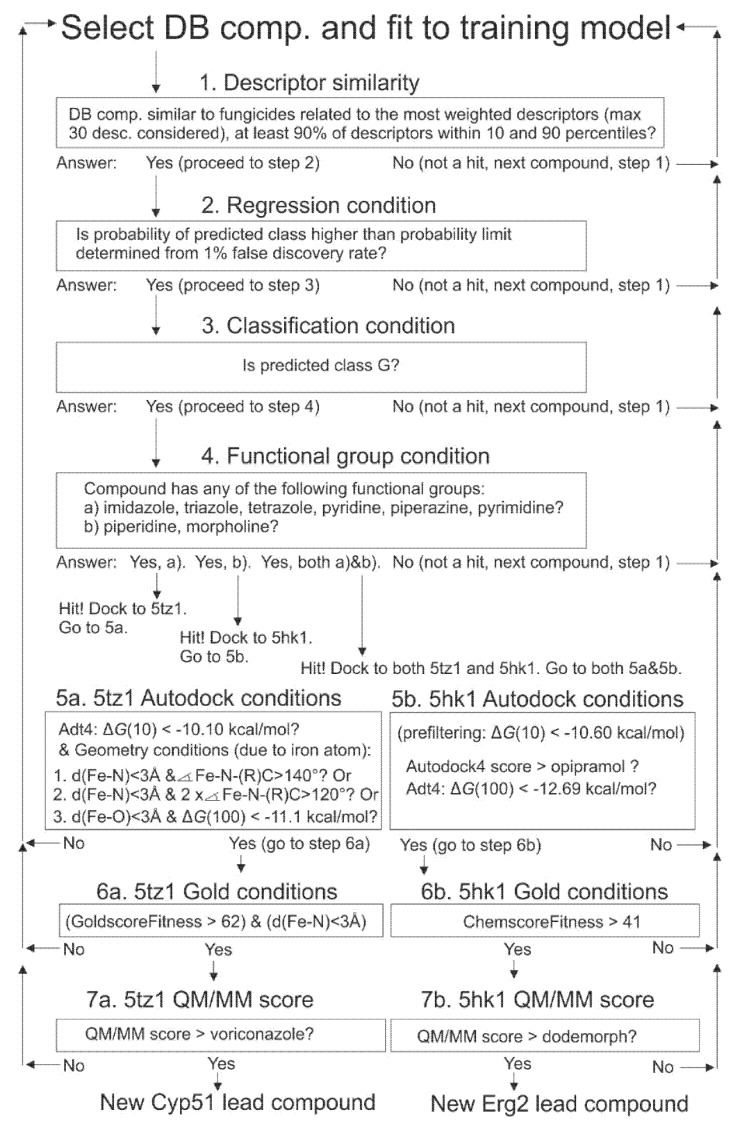
Approach I. In silico drug repurposing process for each compound from the Drugbank database, using the criteria set above. From the molecular descriptors and mode of action working (MOAW) training set, we established models for QM//MM calculations in the target protein. In addition, see approach II (differing only in the first three steps from approach I).

To do so, in the first part of the study (1), the filtering of Drugbank is not easy without a well-established large dataset of non-antifungal compounds. Since there is no such dataset of non-antifungals sufficiently large for Drugbank diversity, we decided to construct the filtering design with two different approaches. In the first approach (I), a MOAW set was enlarged with a non-fungicide set from [3] and reduced for the compounds that were shown to exhibit fungicide activity. This set was used to train a number of different machine learning models. The filtering was carried out under the following criteria: (a) selected hit compounds have to comply with the limit values of the most weighted molecular descriptors of thefungicide data set and (b) the confidence of predictions had to satisfy the false discovery rate criterion of classification models (a very strict limit of 1%) in order to filter out non-antifungal compounds. In this first approach (I), a number of different class discriminant analyses were considered so that the joint model predicted the correct class (G) of each considered Drugbank compound with a high precision. In the second approach (II), 1500 compounds were randomly selected from the Drugbank data set with the assumption of all being non-class G fungicides and then merged with the rest of the MOAW compounds. In contrast to approach (I), we opted here for a regression-based modeling of the target G class. The constructed model was then used to predict the class for the rest of the Drugbank dataset. Those Drugbank compounds predicted as belonging to G were potential hits. The assumption that the set of compounds extracted randomly from Drugbank consisted exclusively of non-fungicides was of course only roughly statistically valid. By constructing a large number of models with different random non-fungicide sets and testing against the remaining compounds from Drugbank, we obtained a larger number of predictions for each Drugbank compound. We combined these predictions, and only those compounds that had a significant number of positive predictions (above the threshold) were considered as potential class G fungicides. Finally, both approaches had to be compared. Furthermore, all compounds classified to group “G” had to contain at least one chemical functional group attributed to either a G1 characteristic for the Cyp51 protein MOA group (see above which; e.g., imidazole group) [1] or a G2 characteristic for the Erg2 protein MOA group (see above which; e.g., piperidine group). Only then was compound regarded as a hit compound and submitted for further molecular docking studies for in silico verification. The exact details of our methodology are displayed in the following article sections (see also later—Scheme 1).

## 2. Materials and Methods

### 2.1. Data Set

The whole sample data set comprised of 215 (FRAC) MOA Code List 2019 [1], plus 30 additional fungicide compounds with chemical groups belonging mostly to imidazoles and triazoles—i.e., with a known mode of action making up a 245 MOAW set of fungicide compounds (Supplementary Table S2, with references therein).

The variable set comprised of 1D and 2D Padel Descriptors [13]; altogether, 1444 descriptors were considered. The number of descriptors was reduced before obtaining a classification model either with uninformative variable elimination plus partial-least squares regression or with a selection of the most important variables using random forest and correlation limitation (see below).

The list of all calculated molecular descriptor groups is mentioned in Supplementary list 1.

From a complete database of ≈13,000 Drugbank drugs, we considered in our study “all structures” given in the “open structures”—structure-data file (SDF) format of 9680 compounds (Table S3a) (see Supplementary file “open structures.sdf” (downloaded on 02-04-2019)). Due to the exclusion of molecular size (n(C atoms) ≤ 40, see why in Supplementary Comment 1) and metal cations, we finally considered 8172 Drugbank compounds with all 1444 non-missing variables (the complete list of considered compounds with Drugbank IDs, CAS no., and smiles in Table S3b). To reproduce our Drugbank dataset, follow the details in Supporting Instructions Note 1.

The first filtering approach (I) is based on solving three different classification problems and datasets: (Ia) 11-class problem for discriminating between 11 FRAC MOA classes of fungicides (245 compounds); (Ib) 3-class problem for discriminating between the most abundant classes C and G and all the rest of the MOA classes (also 245 compounds); and (Ic) 4-class problem in which the 3-class model is extended to the class of 131 non-antifungal compounds from [3] (altogether 376 compounds).

The second approach (II) is a regression-type problem (with dummy variables, “0” and “1”) with only two class models considered. One class denotes fungicides belonging to class G (labeled as “1”), and the other class compounds not belonging to class G (labeled as “0”), including both fungicides not belonging to class G (but belonging to any other class) and non-antifungals. This second approach (II) was constructed from 245 MOAW, plus 131 non-fungicides (Table S1) and 1500 non-constant randomly selected compounds from 8145 Drugbank compounds ascribed as “non-antifungals”, making altogether 1876 compounds. Again, the 1500 were randomly selected from 8145 compounds and not from 8172, because within the 8172 compounds there were already 27 known class G fungicides that were removed prior to the random selection.

### 2.2. Data Preprocessing

We started data pre-processing by first removing uninformative variables (those having very few non-zero values; i.e., less than the number of classes). The values of the original variables were scaled and mean centered. After data cleaning and pre-processing, the final data tables of both the MOAW set and the Drugbank set did not contain any missing values. In each of the problems solved (Ia, Ib, Ic, and II), we used one third of the compounds as the test set and 2/3 of the set as the training set (see Table S2). For example, for approach Ib there were 163 training compounds, while for approach II there were 1251 training compounds.

### 2.3. Uninformative Variable Elimination Partial-Least Squares Discriminant Analysis (UVE-PLS-DA)

A procedure of highest covariance between the matrix of the mean-centered and scaled descriptors, ***X***, and the matrix of MOA designation (***Y***) where each column represents one class and with dummy variables (0 being not belonging and 1 belonging to the class) was carried out. We obtained in several steps the matrix of the column vectors of the regression coefficients, ***B***, on the training set, which linearly relates the descriptors and the MOA; ***Y*** = ***BX + E*** (with ***E*** being the error matrix). This procedure is called partial-least square discriminant analysis [14].

Uninformative variable elimination (UVE) was used to filter noisy variables, which reduced the number of variables to only a few hundred or fewer variables. That was done using the stability values for each variable (i.e., each descriptor value), defined as the ratio of the mean regression coefficient of the corresponding variable and its standard deviation in the cross-validation procedure. Through the cross-validation procedure, the regression coefficient for each variable varied as different samples were left out [15]. So, when the procedure ended, every descriptor had its mean and standard deviation, and the variable’s stability was the ratio of its mean and standard deviation. When, for all variables, the stability values had been determined, then the stability cut-off was set to remove all the variables whose stability was lower than the cut-off stability. The higher the cut-off was set, the more variables would be excluded. For that reason, the cut-off level could be optimized. Using cross-validation, the optimal cut-off stability value was determined according to the highest percent of correct classification (PCC) among all other cut-offs. That was simple in the case of the ordinary UVE-PLS regression procedure used in approach (II) for a single Y column with dummy variables. However, in approach I, there were more classes used for the discriminant analysis. With more classes, each variable had different stabilities for each corresponding class (i.e., the number of stability values equaled the number of columns in the ***B*** matrix); then, the variable stability was selected as the median among the stability values of all the classes in the corresponding model [16]. For the case of four classes, the stabilities of the second-lowest class were taken.

The optimal number of latent variables (LVs) used for building the model was determined with the highest classification accuracy of cross-validation on the calibration set. The percent of correct classification (PCC) was used as the classification accuracy for both the training and cross-validation procedures and the validation set. The upper limit of the number of LVs was initially set to be 20 in each case.

In approach I, leave-one-out cross-validation (LOO) was performed all the time; while in approach II, leave-10%-out (i.e., 10-fold) cross-validation was carried out. This applies for all methodologies in the first machine learning part of the study.

### 2.4. General Rules for All Regression Procedures in Classification

For a tested sample, to be predicted to a class, the highest obtained *Y* value among all columns (i.e., classes) predicts belonging to that corresponding class. In the case of only one *Y* column (as in approach II), if a prediction is ≥0.5, then the corresponding sample is classified as belonging to G-class fungicides. These rules apply for all sections (above and below).

### 2.5. Forward Stepwise Multilinear Regression Procedure

Forward Stepwise Multilinear regression (one-by-one addition of variable) was carried out only for the first approach (Ia-Ic), not for the second (II) (so ***Y*** and ***B*** were matrices with the number of columns equaling the number of classes). The regression was performed on the raw data variable preprocessed data (stepwise multilinear regression (FS-MLR)) and on that subset of variables (i.e., descriptors) already selected by performing UVE-PLS filtering (of noisy variables) on the corresponding number of used classes (11, 3, or 4), which will be called UVE-PLS-FS-MLR, where again UVE-PLS regards only the preselection of the original variables. This (UVE-PLS-FS-MLR) resulted in a much faster algorithm than FS-MLR since, at the start, FS-MLR dealt with more than 1000 variables. A forward stepwise procedure was designed for merging each new descriptor variable with the prior variable that resulted in the highest possible increase in PCC(CV), with a limit of up to 30 descriptors. The best FS-MLR model was the one with the highest PCC(CV) among all other 30 models, with special attention that if the addition of any new variable results in a decrease in PCC(CV) then the procedure stops.

### 2.6. Random Forest Procedures

Random Forest (RF)-based variable selection was performed using the randomForest R package [17] in program R [18]. Two different procedures were used, classification and regression.

### 2.7. UVE-PLS-Random Forest (UVE-PLS-RF)

Firstly, for each corresponding class, the already-filtered set of reduced number of the original variables obtained from the UVE-PLS(-DA) was used for RF procedures. This is called UVE-PLS-RF. On this reduced set of original variables, the RF cross-validation procedure was carried out to determine the optimal number of trees for the RF. Using the model with the optimal number of trees based on the maximal cross-validated PCC, the PCC of the test set (PCCte) was calculated. The optimal number of trees determination was carried for the first approach (models Ia–Ic), but for the second approach (II) the number of trees was always 300, as any different number of trees did not affect the results significantly. In approach I, classification was carried out for RF, but in approach II, regression was carried out.

### 2.8. Forward Stepwise Limited Correlation Random Forest Procedure

In this procedure, classification was performed with RF for approach I, while regression was performed with RF for approach II. Firstly, the random forest cross-validation was used on all the (raw, preprocessed) variables to determine the optimal number of trees for the random forest (except for approach II, where a fixed number of 300 trees was used). Then, the variables were sorted based on the “meandecreaseaccuracy” RF coefficients for approach I and on %IncMSE for approach II. The first top 100 variables with the highest “meandecreaseaccuracy” (or the highest %IncMSE) were taken into further account, while all the other variables were discarded. The correlation matrix was built between these 100 variables and the longest sequence of mutually uncorrelated variables, i.e., variables only correlated below a certain threshold were selected, while other variables were discarded. The correlation thresholds were: 0.9, 0.8, 0.7, 0.6, and 0.5. The RF procedure then started creating regression/classification models by merging variables one by one through that (uncorrelated) variable sequence of decreasing “meandecreaseaccuracy” (i.e., %IncMSE) by accounting for at least the top 12 variables, and the model with the highest classification accuracy was selected based on the PCC of the cross-validation. This stepwise procedure ended if, after 12 variables or more, the addition of another variable did not improve the model’s cross-validation accuracy (5-fold cross-validation was utilized in this step). This procedure is called Forward Stepwise Limited Correlation Random Forest (FS-LM-RF) regression, and is considerably faster than the FS-MLR procedure.

### 2.9. Selection of Drugbank Hit Compounds, Approach (I)

All the 11-class (Ia), 3-class (Ib), and 4-class (Ic) models obtained using UVE-PLS-DA, UVE-PLS-RF, FS-MLR, UVE-PLS-FS-MLR, and FS-LM-RF were used for the prioritization of 8172 DrugBank compounds, with two criteria: (1) The first criterion was that the DB compound had values for at least 90% of the most (regression) weighted selected molecular descriptors (up to, at most, 30 descriptors for UVE-PLS-DA) between the 10 and 90 percentiles, considering the corresponding descriptor values of the MOAW training set. The second criterion (2) was that those DB compounds that had passed the first criterion had classified the MOA probability rate higher than 1% false discovery rate probability. A 1% false discovery rate was calculated as the probability rate of falsely classified top 1% maximum classification, taking into account both the cross-validated training fungicide MOAW samples and the tested MOAW samples.

When both criteria (1) and (2) were met, then all results of all the obtained models concerning all the class analyses were merged, and only those compounds classified as MOA group G from the Drugbank database were taken into further consideration. The others were discarded, which was the third step in the criteria elimination procedure, as depicted in Scheme 1. The basis for this kind of approach was to select only those compounds with a close similarity to antifungal compounds regarding the selected molecular descriptors and, at the same time, with a high likelihood of belonging to the “G” MOA class of antifungal compounds. That was the basis of approach (I) for the determination of novel antifungal compound hit molecules. The fourth (4) and last step was that if compounds that passed step three had any of the chemical functional groups that the fungicides of G MOA possess—which are triazole, imidazole, pyridine, pyrimidine, piperazine, morpholine, piperidine, tetrazoles and spiroketal-amines. If selected compound does not have any such group then it is discarded. We even considered some conjugated and/or substituted derivatives of these functional groups. Compounds that survived all these steps were regarded as “hit compounds” for this approach (I) and taken into further consideration for the docking studies (Scheme 1).

### 2.10. Selection of Drugbank Hit Compounds, Approach (II)

**Scheme 2:**
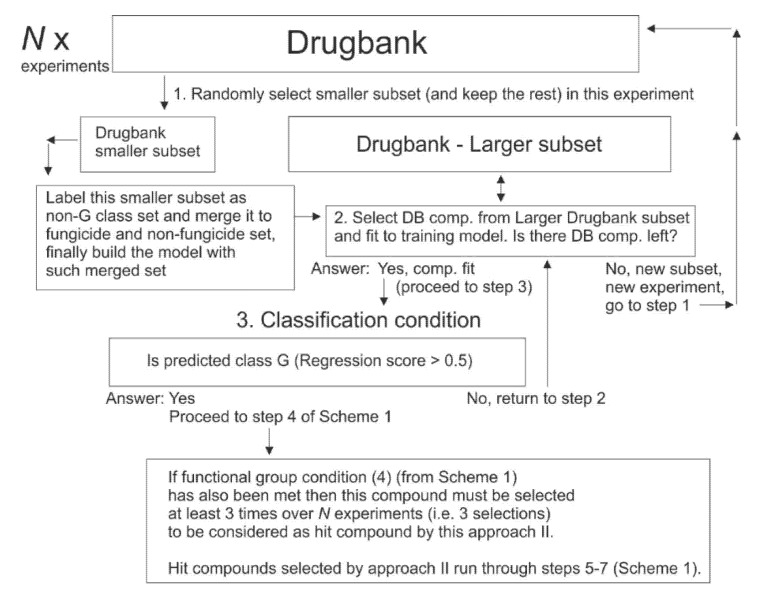
Approach II. In silico drug repurposing process, alternative machine learning steps (1–3). The rest of the steps, from step 4, are the same as in Scheme 1.

Among the 1876 compounds, 1251 were training and 625 test compounds. Among these 1251 training compounds, there were only 58 positives “1”. The models were obtained on the training set and validated on the test set. Altogether, 35 UVE-PLS, 20 UVE-PLS-RF, and 30 FS-LM-RF experiments (summed 85) were carried out. Each experiment was conducted with 1500 differently randomly selected compounds from 8145 Drugbank compounds. We assumed that each of these compounds wasn’t a class G fungicide. Finally, the training models were extrapolated to the remaining 6672 compounds. These 6672 were only used for hit compound determination. The number was 6672 because 8145 − 1500 = 6645, 6645 + 27 = 6672. We used “+27” because there were 27 class G fungicides of the Drugbank database that were added to the larger set as a test and were finally recognized by the model. These 27 were omitted before the random selection of the smaller dataset but then added to the larger dataset (Scheme 2). All the Drugbank compounds predicted to be “1” at least three times among these 85 experiments were selected to b hit compounds (Scheme 2). Why more times than one? This was for protection against the possible (but unlikely) accidental random selection of too many confounding class G fungicides into the training set. Why “three”? It was calculated as a rounded 5% of the number obtained by multiplying the total number of the approach II experiments (which was 85) with the ratio of the larger Drugbank subset (6672 compounds/total Drugbank dataset of 8172 compounds). Therefore, 0.05 × 85 × (6672/8172) = 3.47, and a round of 3.47 equals 3. The descriptor similarity criterion was not considered because it would not be a similarity to general antifungals (as in approach I); it would only be a similarity to class G fungicides, which wasn’t necessary in this binary case where the regression model specifically discriminated between “1” (G) and “0” (non-G) while taking into account 1000 Drugbank compounds, which covered enough structural diversity in our opinion. Outliers as false negatives, i.e., false “0”, were possible in the model creation. A few outliers hardly affected the model among 1251 training compounds. However, because of possible outliers, the false discovery rate criterion also wasn’t applicable, since even a single outlier could be falsely predicted as a “false positive” although it was actually a true positive, and then the false discovery rate based on the “false prediction” of truly correctly predicted compounds would turn out to be erroneous. Finally, the compound with three selections had to possess any of the chemical functional groups that fungicides of G MOA possess (see section above); only then was such a compound predicted to be a hit compound (Scheme 2).

The separation of all the obtained hit compounds was based on the G MOA subclasses, G1 and G2, based on the target protein. The G1 subclass had a *Candida albicans* Cyp51 protein target (see next section), and comprised the selected hit compounds with pyridines, piperazines, pyrimidines, triazoles, triazolinthiones, or tetrazoles. The G2 subclass had (homology approximated) a human sigma 1 receptor protein target (see next section) and comprised the hit compounds with morpholines, piperidines, and spiroketal-amines.

### 2.11. Docking Studies

#### 2.11.1. Autodock4 Prefiltering Docking Phase

The target proteins were taken from the Research Collaboratory for Structural Bioinformatics (RCSB) Protein Data Bank (PDB) as 5tz1 for the *Candida albicans* Cyp51 protein and 5hk1 for the human sigma 1 receptor, as its structure concerning pharmacological similarity is very similar to Erg2 in yeast, which catalyzes 8,7 sterol isomerization based on the overall 30% sequence homology, 66% homology in the putative S1R ligand-binding domain [19], and the pharmacological correlation coefficient of 0.83 [20].

For the 5tz1 and 5hk1 proteins only, chain A was considered with removed ligands and water molecules so that the protein was in a ready form for ligand docking. For the 5tz1 grid coordinate, the center was x = 70.136, y = 65.316, z = 4.711 for 5tz1. For the 5hk1 grid coordinate, the center was x = 12.0, y = 38.0, z = −36.0 (which are roughly arithmetic means of the coordination coordinates of the used ligand PD144418).

Additional protein targets for the Cyp51 protein were considered, with only chain A analyzed and stripped of all ligands and water molecules, such as human Cyp51 taken from Drugbank 3ld6 (coordinate center: x = 44.78, y = 4.141, z = 2.828), *Saccharomyces cerevisiae* 5eab (same coordinate center: x = 4.885, y = 10.024, z = 95.771), and 5eqb (coordinate center: x = 15.896, y = 11.719, z = 18.001). The obtained docking scores for each of these targets for 64 fungicides were tested for mutual correlation between these targets and the 5tz1 target.

The 5tz1 protein contained protoporphyrin with hexacoordinated Fe. The charge +2.0 on the Fe was used differently to the Autodock4 default charge, as was also used in a study by Chaskar et al. [21]. The explanation as to why we utilized this charge, we provide in supporting information note 1.

All the Cyp51 and Erg2 fungicides along with all the obtained hit compounds were analyzed with the Autodock4.2 program. The prefiltering docking phase consisted of discriminating between all the tested compounds with the lowest binding energy below the certain energy cut-off with 10 ga runs. For the 5tz1 protein, this very specific “cut-off” was determined as the score obtained for oteseconazole (VT1161), which was the experimentally bound ligand in 5tz1. For the 5hk1 protein, this very specific cut-off was determined as the score obtained for PD144418, which was the experimentally bound ligand in 5hk1. For each corresponding target, only those hit compounds ranked higher than the determined cut-off score were merged and considered further for the Part 2 docking phase, while all other hits were discarded. 

#### 2.11.2. Docking Phase

**Figure 1:**
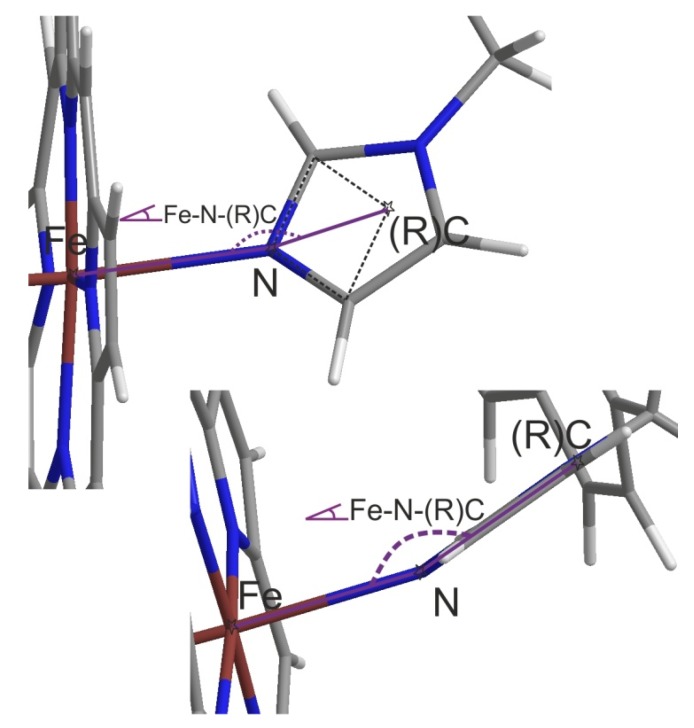
Definition of the Fe-N(R)C angle. ′(R)C′ is the in-plane ring coordinates spanned by the vector sum of the two neighboring atoms to the N coordinated atom. In the specific case of nitrile bonded ligand (Fe-N≡C), the angle is the Fe-N-C angle (without ′(R)′).

Regarding the 5tz1 protein, hit compounds that passed prefiltering were submitted to the new Autodock4 docking study where, instead of 10 genetic algorithm runs, 100 runs were used. For these compounds, Autodock vina was also carried out. Autodock vina was performed using 20 binding modes and an exhaustiveness of 500, as had already been used in a similar protein-ligand system in literature [21]. The grid size was in all cases 34 × 34 × 34 Å, with the already-mentioned-above grid centers. Autodock vina was used only for 5tz1. The goal was to achieve any conformation among 100 conformations for Autodock4 and, in addition, 20 conformations with Autodock vina, with either a nitrogen (N) or oxygen (O) atom within a 3 Å distance from the iron atom in the 5tz1 protein. At first, the search was based on whether or not nitrogen was within a 3 Å distance. If not, then there had to be any conformation with the O atom within Å stronger than 11.1 kcal/mol. If that still wasn′t the case, we rejected that hit compound. If there was a nitrogen atom within a 3 Å distance, special attention was paid to the Fe-N(R)C angle between the iron atom, the nitrogen atom, and the azole or pyridine or pyrimidine ring center (defined by the vector sum of the two neighboring ring atoms of bound N to Fe; see later Figure 1) to see which angle was formed when explaining the overlap of the in-plane azole nitrogen orbital of the donating nitrogen-free electron pair and electron-accepting free iron d orbital. That angle cut-off will be determined later.

Concerning the 5hk1 protein, all the hit results, after passing prefiltering, were submitted to an additional (second) energy cut-off in Autodock4, this time with 100 ga runs (instead of 10) because most of these compounds were close to Glu172. The cut-off value was determined concerning the minimum energy conformation obtained for opipramol, which, in terms of absolute values, had the highest cut-off value among all the compounds well known in the literature for the Erg2 protein [20] considered in this study. All the hit compounds with higher scores than opipramol were submitted to the last and third QM/MM minimization stage.

All the Autodock4.2 studies were run with genetic algorithm defaults (e.g., the maximum number of energy evaluations was 2,500,000 and the maximum number of generations was 27,000). The grid size was in all runs 90 × 90 × 90, with spacing at 0.375 Angstroms. The final result in this part 1 docking phase was represented as the lowest energy docked conformation. Again, in this (screening) phase just 10 ga runs were used.

#### 2.11.3. Part 3 QM/MM Studies

All the QM/MM studies were performed in ORCA version 4.2.0 [22]. Different procedures were carried out for 5tz1 and 5hk1. However, in both procedures the functional and dispersion correction used was B97-D3 [23,24], with the default ORCA grid used during the optimization phase. Prior to the ORCA minimization, both the proteins (which only included chains A) were submitted void of all ligands (including protoporphyrin) to a VMD program where Protein Structure Files (PSF) and PDB files were constructed for the ORCA input. The ORCAFF.prms file for both the 5hk1 and 5tz1 proteins were constructed using a Charmm force field (via Nanoscale Molecular Dynamics (NAMD)), while the ligand force field files were initially created and later merged with the protein while retaining all the ligand coordinates from the Autodock4 docking log (DLG) output file [25]. The optimization runs were set with the ORCA defaults, employing the keyword “Opt”. The Polarizable Continuum Model (PCM) model was used in all the calculations with “%cpcm” as the keyword and with epsilon 80 and “refrac” 1.33 (water conditions). After the convergence of the ligand-protein complex, the sole ligand was optimized, with initial input coordinates the same as those obtained for the converged protein-ligand structure.

For the 5tz1 protein, firstly, the hydrogen optimization was performed using the “!L-OptH” option for the protein amino-acids and the heme. Heme is a complex in the active center of 5tz1, consisting of an iron ion coordinated to a protoporphyrin ring acting as a tetradentate ligand. The hydrogen atom on the sulfur atom coordinating the heme from the cysteine residue was manually removed, making an S^–^ anion that coordinated the iron atom from the opposite side of the ligand site, as already noted for cytochrome P-450 [26]. CH_2_-S^–^ was put into the QM region along with the central part of the heme and each considered ligand molecule. The water molecule was bound to iron in the low-spin state of Fe^3+^, as was the case with the azole antifungals [11,26]. Thus, Fe was treated as +3 and the overall spin (2S+1) equaled 2. For the protoporphyrin nitrogens –2 with the negative cysteine sulfur atom –1, without protoporphyrin side chains in the QM region (two COO^–^) groups, the whole active center (excluding the ligands) had a zero (0) total charge in the QM region. The whole protein including the heme was treated as inactive, except for some parts of some residues mentioned in Reference [11] that were reported to form contact with oteseconazole; for more details on active and QM atoms, see Supplementary Table S4. In the optimization phase, the used basis set for the QM atoms was 3-21G, except for all sulfur (S), oxygen (O), and nitrogen (N) atoms, for which the basis set was 6-31G*, while the Wachter basis was used for the Fe atom (i.e., {611111111/51111/311/1}). An auxiliary basis used was set in the input as SARC/J (for definition see Ref. [25]). With the optimization converged for the protein-ligand complex, the same was done for the sole ligand, and then the final single energies for the complex, sole protein, and sole ligand were estimated using a def2-TZVP basis, in addition to the basis set superposition error correction employed by the input keyword “GCP(DFT/TZ)” [25] and with Grid6. With the geometry optimizations converged, the frequency calculations were estimated at zero-point energy (ZPE) and a thermochemistry at 298 K.

For the 5hk1 protein, the whole protein (chain A, void of all water molecules and ligands) was treated as inactive without any QM atom considered (i.e., the whole protein was the MM part); only the ligands were the QM and active part. For the ORCA geometry optimization runs, a def2-SVP def2/J basis was used, including frequency runs on the optimized structures. The final energy evaluation on the QM/MM optimized geometry was done using the same level of theory, except for using the larger grid, Grid6 (instead of the default grid), and with a geometry counterpoise correction invoking the command in ORCA “GCP(DFT/SVP)”.

Different ionization states for both the 5tz1 ligands and 5hk1 ligands were extensively taken into account. For 5tz1, the final results presented are those obtained for the neutrally charged species. The results obtained for the charged ligands did not change any important conclusion in this work, but are still presented in the supporting information (Supplementary information note 2).

For the 5hk1, the ligand ionization states considered were those existing in the water solution at pH 7.4 (cations) and (artificially) neutral uncharged species. Consequently, two comparisons between the literature ligands and the hit compounds were made, one between both ions at pH 7.4 and the other between the (artificially) neutral species. The artificially neutral systems (without added counter-ions, but with the addition of H atoms) were already considered in order to prevent an irrationally high estimation of the binding energy [27].

### 2.12. Molecular Docking Verifications With Gold Program

For Gold docking [28] for the 5tz1 target, the same grid center was used as in Autodock4 and all the atoms were selected within a 30 Å distance using the detect cavity option. A GoldScore was used with the “goldscore_p450_csd template” loaded. The genetic algorithm settings were user-defined with the default initial values, except an annealing Van der Waals distance of 4.0 Å (instead of 6.0 Å) and a 1.0 Å H bond distance (instead of 3.0 Å), which was necessarily requested by the program to be within 0.1–1.0 when using the GoldScore. In opposition to Autodock4 and Autodock vina, which used a rigid protein structure, all the experiments were conducted with the following 10 amino-acid flexible sidechains selected for the 5tz1 protein and one torsion angle of 10 degrees’ increment: Tyr64, Tyr118, Thr122, Phe126, Tyr132, Phe228, Thr311, Phe380, Tyr505, and Ser507. For the 70 literature fungicides and 37 G1 hit compounds, there were 20 ga runs set, while for the 7 hit ligands that passed all 7 steps, a repeated experiment was conducted with 100 ga runs.

For the 5hk1 protein and for the 5tz1 target, for all runs, the same grid center was used as in Autodock4 and all the atoms were selected within a 30 Å distance; detect cavity was on. A Chemscore was used, with the “chemscore_kinase template” loaded. The genetic algorithm settings were automatic. In contrast to Autodock4 and Autodock vina, which used a rigid protein structure, all the experiments were conducted with the following 10 amino-acid flexible sidechains selected for the 5hk1 protein and one torsion angle of 10 degrees’ increment: Met93, Tyr103, Leu105, Phe107, Ile124, His154, Glu172, Ile178, Leu182, and Tyr206. The experiments were conducted for all the literature fungicides, literature-important sigma 1 receptor ligands, and all Erg2 hit compounds that passed step 5b (Scheme 1).

## 3. Results and Discussion

### 3.1. MOA Classification Approach (I)

The MOA Classification and Drugbank selection results of UVE-PLS, UVE-PLS-RF, FS-MLR, and UVE-PLS-FS-MLR (a) are displayed in Table 1, while results of FS-LM-RF are displayed in Table 2. The baseline statistical expectation PCC (see below Table S2) for 11 classes is 35.1% (majority class percentage), for 4 classes 34.8%, and 40.8% for 3 classes. The results for all the methods are well above the baseline, and when the different methods are mutually compared, there are no significant differences between either PCC(CV) or PCCte. Concerning PCCte, we find all these results with the obtained hits to be acceptable for further docking evaluations. Overall, the applied methods appear to classify the MOAW set with an acceptable level of accuracy. What is worth noting is that four class models generally produced a low number of hits for many variable methods (UVE-PLS preselected), which is because that non-fungicide class was included in the classification procedure, while the 3- and 11-class analysis did not have a non-fungicide class but only the already-described prioritization rules. On the other hand, for FS-MLR and FS-LM-RF a significant number of hits were also produced for the 4-class models.

Concerning the selected descriptors, among methodologies with the same or less than 30 overall selected descriptors (MLR(6) and FS-LM-RF(15), 21 methods), full lists of the selected descriptors with the number of selections are in Supplementary Table S5a–c.

Regarding the selected compounds of the Drugbank database (Table 1 and Table 2), the full list of all the selected compounds is presented in Supplementary Table S6a. Altogether, 147 different hit compounds from Drugbank were selected. From these, 96 hits belonged to the G1 subgroup, 36 to subgroup G2, and 15 to both G1 and G2 (in total, this is 111 in G1 and 51 in G2). Additional comments are given in the Supplementary information (e.g., we selected pure triazoles but also many conjugated derivatives or other derivatives; also, in this selection stage no attention was given to the geometry of the active site and the possible steric hindrance of the hit compound (i.e., ligand) reactive functional groups).

The 8172 considered compounds (Table S3b) from Drugbank also contained 27 already-known fungicides, and these were also predicted “as hit compounds” (with only one exception—albaconazole) by our classification models (Table 1 and Table 2, last column, number in brackets gives the number of all such defined hits for each methodology). We will mention now these fungicide hit compounds (with the number of hit selections for the same compound given in brackets): fenpropidin (5), naftifine (1), terbinafine (3), tolnaftate (2), miconazole (15), bifonazole (3), terconazole (2), fluconazole (1), ketoconazole (4), sulconazole (5), efinaconazole (9), butoconazole (6), oxiconazole (5), econazole (16), (S)-econazole (16), isavuconazole (4), sertaconazole (11), voriconazole (7), luliconazole (2), ravuconazole (4), albaconazole (0), levoketoconazole (3), oteseconazole (i.e., VT1161) (1), dapaconazole (3), isoconazole (11), and tioconazole (13). The overall sum of the numbers in brackets gives 152 total selections. Since there were altogether 27 models used in the scoring (15 × FS-LM-RF plus 6 × MLR plus 6 × UVE-PLS(DA or RF) = 15 + 6 + 6 = 27) and there were 27 possible fungicides to select, the maximum number of possible selections of these fungicides from Drugbank is 27 × 27 = 729. 152/729 = 20.85%. Thus, the average percent of correct classification of the known fungicides from Drugbank with this classification approach (I) is 20.85%. In addition, oteseconazole, isoconazole, tioconazole, and dapaconazole were successfully selected as hit compounds from Drugbank, although these fungicides were not part of the MOAW set (Table S2). It is important to note that some of these selected compounds (e.g., miconazole (15)) were part of the test set that did not participate in building the models. This means that this classification approach (I), when all results are taken into account, could yield new hit compounds (excluding the literature fungicides) among 147 of them that could have a relatively high probability to be confirmed as novel antifungal agents with the specific mode of action.

### 3.2. Two-Class Ensemble Regression-Approach (II)

Table 3 represents the final results of the two-class regression approach (II). The results are averaged over 85 experiments/models (all the most important results are shown in Supplementary Table S6b). To understand the PCC(CV) and PCCte columns, the baseline statistical expectation for the two classes is 95.36% due to 1193 negatives and only 58 positives in the training set, with a similar ratio of positives to negatives in the test set. The Percent of Correct Classification of True Positives for the test set (PCCTP(te), Table 3) and is fairly high (baseline expectation is only 4.6%). For these regression procedures, we have also calculated a root-mean-square error of prediction. The average obtained on the test set for UVE-PLS was only 0.15, implying stable models. The PCC(27 fung) is the correct classification percentage of the 27 already-known fungicides from Drugbank and, as can be seen, on average all the methodologies for this approach (II) obtained 85.27%. This result is well above the prior classification approach (I) (see prior section above) that obtained only 20.85%. Besides, this approach (II) seems to select only 37 compounds with three selections. Among these 37, 20 of them coincide with approach (I) and 17 of them are new hit proposals (16 G1 and 1 G2). If only one selection was necessary, then the overall hit compounds would amount to 100 (with 813 selections in the total selections). Among these 813 hit instances (i.e., selections), 581 (≈71%) selections belong to those hit compounds selected by approach (I) (Table S6b). For that reason, although approach (II) seems to generally outperform approach (I) in the classification success rate, the similarity in predictions between these two approaches is reasonably high enough to conclude that the compound hits selected by approach (I) can all be included in further docking studies, as can the additional 17 hit compounds for consideration selected by approach (II) (see Table S6b).

### 3.3. Docking Study

#### 3.3.1. Autodock Results and Comments

##### 5tz1 Docking

Information about the minimum energy docked scores for the known fungicides is given in Table 4 and more in Supplementary Table S7. Here, the evaluation of the docked structures for each fungicide was made according to (a) the minimum Gibbs free energy (Δ*G*) conformation of all 100 conformations considered (in the latter text Δ*G*(100)), (b) the minimum energy conformation for the subset of conformations where the distance Fe-N < 3 Å (in the latter text Δ*G*(<3Å)), and (c) the maximum angle (Fe-N-(R)C angle, Figure 1) conformation for the subset of conformations with a distance Fe-N < 3Å (with binding energy given in Table S7, Δ*G*(max α)). Regarding these three energies, the following relation is valid: Δ*G*(100) ≤ Δ*G*(<3Å) ≤ Δ*G*(max α).

Besides taking into account the nitrogen vicinity and angle, we took into account which nitrogen was coordinated, with the accent to specific nitrogen, which for imidazoles was N3 imidazole nitrogen, for triazole was N4 triazole nitrogen, and for tetrazole was N4 position nitrogen, which is already established to form a coordinated bond with protoporphyrin iron [11]. The selection of the most favorable Fe-N-(R)C angle is a priority for the QM/MM minimization for a structure to converge with an Fe-N bond, since it is necessary to establish an as good as possible geometry input overlap between the in-plane azole nitrogen orbital of the donating free electron pair from nitrogen and the electron-accepting free d orbital from iron. The simple use of minimum energy conformation among all 100 runs does not make too much sense for the best QM/MM geometry input, because the scores of the Autodock4 program do not take into account the quantum mechanical and polarization effects of the iron atom.

In comparison with the literature, the Δ*G*(100) for the most frequent fungicides (posaconazole, ketoconazole, miconazole, fluconazole, voriconazole, clotrimazole, itraconazole, and VT1161) is correlated with the experimental inhibitory effect of these drugs on *C. albicans* Cyp51 (values taken from Reference [11]) with Pearson′s *R* of 0.742. In other publications [29], between clotrimazole, fluconazole, itraconazole, ketoconazole, and voriconazole, fluconazole had the weakest experimental binding for both *C. albicans* and human Cyp51, as was determined also with Autodock4 in this study. In the same study [29], itraconazole and ketoconazole had the lowest dissociation constant, *K*_d_, for human Cyp51, while only voriconazole had a lower *K*_d_ for *C. albicans* Cyp51 in this study, while Autodock4 ketoconazole and itraconazole had the strongest bindings to *C. albicans* Cyp51 among these five considered antifungals. The *K*_d_ for ketoconazole for *C. albicans* Cyp51 was determined to be ≈12 nM, while for propiconazole it was ≈38 nM [29]. Here, for propiconazole and 10 ga runs, the *K*_d_ was 46 nM. Finally, attempts to dock ligands to the Cyp51 protein have already been made [30] where the proper orientation of the azole nitrogen has been criticized. This means that geometry imperfections due to, e.g., the angle between triazole/imidazole nitrogen and Fe (which we have addressed), can be expected. In Reference [30] for zebrafish Cyp51, Autodock yielded binding energies of −10.7, −9.7, and −7.4 kcal/mol µM for lanosterol, ketoconazole, and propiconazole, respectively. We calculated a binding energy of lanosterol to the *C. albicans* Cyp51 protein of −11.71 kcal/mol. Among 100 conformations, it did not contain any conformation which included any bond with iron atoms, but that was as expected [31] since the substrate binds differently than inhibitors (antifungals); inhibitors should bind to iron, contrary to the substrate, which may not be the case due to the oxygen mechanism already described [31]. In conclusion, our results obtained with Autodock4 for 5tz1 seem to be comparable with the literature and might be used for a rough assessment of the binding affinity of new hit compounds.

In addition, in this study we established a high correlation for 64 docked antifungals between 5tz1 and 3ld6 (*R*^2^ = 0.917), 5tz1 and 5eqb (*R*^2^ = 0.905), and 5tz1 and 5eab (*R*^2^ = 0.921).

For the ligands established to contain a bond with Fe other than with nitrogen (i.e., other than Fe-N), we give their corresponding binding affinities: aliconazole Fe-Cl with −9.68, azaconazole Fe-Cl with −7.89, quinconazole Fe-Cl with −9.30, pyrifenox Fe-O with −9.16, pyrisoxazole Fe-Cl with −9.12, terconazole Fe-Cl with −10.84, triadimefon Fe-O with −8.03, triflumizole Fe-F with −6.95, triforine Fe-O with −5.92, uniconazole Fe-O with −7.82, uniconazole-P Fe-O with −7.63 (all values are in kcal/mol, respectively).

Now, all these results have been taken into account and evaluated to determine the filtering criteria for the hit DB compounds. The most important result is for the 5tz1 crystallographic ligand oteseconazole (VT1161), with 10 ga runs and Δ*G*(10) = −10.10 kcal/mol. This will be the prefiltering energy criterion in Scheme 1 for hit compounds and is a little bit more strict than average in Table 4 (−9.43) for N-Fe coordinating antifungals, but is slightly lower than the ΔG(100) for oteseconazole. The results of such prefiltering among the 127 G1 hit compounds (111 + 16, i.e., 16 for approach II) are shown in Supplementary Table S8 (regarding only the ΔG(10) column).

Now, in order to pass to the QM/MM simulations, the hit compounds have to satisfy condition 5a with 100 ga runs in Scheme 1 (at least one of the mentioned criteria). Thus, a hit compound has to contain either N close to Fe (<3Å) with a Fe-N-(R)C angle >140 degrees or N close to Fe (<3Å) with at least two conformations of the Fe-N-(R)C angle >120 degrees. The reasons for these criteria are the following: The average angle value in Table 4 is 150.8° and setting that angle cut-off would be too strict, since even the most potent fungicides like posaconazole or itraconazole would not meet that criterion, so it should be set lower than that. The first round number (in 10°) would be 140° to meet posaconazole and itraconazole. If a Fe-N-®C angle >140 is set then exactly 75% of fungicides (all except the lower quartile) in Table 4 obey this criterion. Still, some important fungicides might not meet this, but if more conformations (i.e., at least two conformations) with the N atom among the 100 conformations are close to Fe with a minimum angle of 120°, we flexibly consider such hit compounds while taking the conformation with the highest possible angle. In addition, we allow other conformations than specific azole nitrogen bound to Fe, but only for oxygen (Cl, F, I, or even S are simply too weak ligands to consider for QM/MM simulations). So, if the hit compound does not have any conformation of N with Fe (<3Å), it at least must have oxygen within 3 Å, but such a conformation must be strong. Thus, we set an alternative additional rule for non-nitrogen, but with dist(Fe-O < 3Å). As in the prefiltering, we have already established −10.1 kcal/mol; we raise the bar in absolute terms by at least −1 kcal/mol, obtaining a cut-off value of −11.1 kcal/mol (Scheme 1) for Fe-O < 3Å.

From these results (Table 5), the compounds that have finally passed to the last QM/MM stage are those for which the column “For QM/MM” is “yes”; all others failed to pass. More detailed results of this docking stage 5a are presented in Supplementary Table S8.

##### 5hk1 Protein

The root-mean-square deviation (RMSD) for the PD144418 ligand between the crystallographic and minimum energy docked structure using Autodock4 is 1.32 Å.

The results of all the Erg2 ligands and literature-important ligands for the 5hk1 protein in Autodock4 (minimum docked energy conformation in kcal/mol) are given for 10 genetic algorithm runs (runs accompanied subsequently with additional analyses with 100 ga are given in brackets): (Erg2 ligands) aldimorph −9.00 (−9.01), dodemorph −10.99 (−10.92), fenpropidin −9.9 (−10.07), fenpropimorph −10.57 (−10.71), tridemorph −8.56 (−9.20), piperalin −9.76 (−9.89), and spiroxamine −9.68 (−9.77). Those literature-important for the σ1 receptor are 4-IBP −12.03 (−12.06), amiodarone −10.83 (−10.37), clomiphene −11.78 (−11.25), emopamil −10.7 (−11.15), fecosterol −11.32 (−11.56), haloperidol −11.77 (−12.01), ifenprodil −11.03 (−11.09), NE-100 −9.33 (−9.89), opipramol −12.87 (−12.69), PD144418 −10.6 (−10.61), pentazocine −8.15 (−8.12), sufentanil −9.5 (−9.80), tamoxifene −11.9 (−11.11), and triparanol −11.74 (−11.83). Among all the considered ligands, PD144418 is a crystallographic ligand of 5hk1 and its score was used as a prefiltering value for the evaluation of hit compounds. The cut-off was used with 100 ga runs but with the top-rated non-hit ligand, which, among all the considered literature ligands, is opipramol (−12.69 kcal/mol).

The full results for all the considered G2 hits docked to 5hk1 are in Autodock4 in Supplementary Table S9. Here, we present only the important results, i.e., only those hit compounds with a minimum docked energy conformation in kcal/mol using 100 ga runs of score with an absolute value higher than the cut-off for opipramol (−12.69), which are DB06555 (−14.22), DB08746 (−13.25), DB08622 (−13.18), DB00637 (−13.64), DB02491 (−14.19), DB07075 (−15.55), and DB12345 (−13.08) (Table S9). Only these compounds passed the Autodock4 5b condition (see also Table S9).

For ten literature important ligands—opipramol, tamoxifen, haloperidol, triparanol, ifenprodil, amiodarone, emopamil, fenpropimorph, pentazocine, and fecosterol—we established a positive Pearson’s correlation, *R*, of +0.864 between the Autodock4 *K*_d_ values determined in this study (when the binding energy in kcal/mol is expressed in *K*_d_ values) and the estimated pharmacophore *K*_d_ values of the same ligands with the Erg2 protein in the work of C. Laggner et al. [20]. This is a promising result. However, regarding fenpropimorph and tridemorph, the literature [32] in contrast to our results for Adt4 predicts a much stronger binding to both the Erg2 and σ1 receptor proteins. For tridemorph with 100 ga runs, Autodock4 predicted a *K*_d_ of 144 nM, while for fenpropimorph it predicted 17.5 nM. On the other hand, for tridemorph the experiment determined a *K*_d_ of 0.09 nM for Erg2 and 0.04 for σ1. Meanwhile, for fenpropimorph, it determined a *K*_d_ of 0.05 nM for Erg2 and only 0.011 nM for the σ1 receptor, which is a considerable difference. The difference between the literature and our results can also be seen with tamoxifen; here, we obtained a *K*_d_ of 1.85 nM, compared to the Erg2 value of 1470 nM and the σ1 receptor value of 34 nM [33,34].

#### 3.3.2. Gold Docking Score Results

Before submitting the optimal conformations from Autodock to QM/MM, an additional molecular docking verification was carried out with the Gold program. We established a Pearson’s *R*^2^ of 0.695 between the GoldScore Fitness and the Autodock4 ΔG(100) for 70 G1 fungicides of the Cyp51 target (Table S10). A similar, although somewhat worse, correlation (*R*^2^ of 0.455) was obtained for the 21 literature ligands of Erg2 target (Table S10). This means that the scores obtained with Autodock4 for these targets roughly follow the pattern of the Gold docking program. In these cases, both the proteins used flexible sidechains (instead of Autodock4 that only used a rigid protein structure). Among 39 5tz1 hit ligands that passed the prefiltering of step 5a, the GoldScore for 38 of them is the same or above a median obtained for 70 fungicide compounds (Table S8). Among seven 5hk1 ligands that passed condition 5b, the Chemscore for six of them is the same or above a median obtained for 21 Erg2 inhibitor compounds (Table S9). Although the hit compounds that passed step 5 (5a and 5b) were considered for the QM/MM, in the next section we show that all the compounds that passed the final QM/MM step also passed the Gold step 6.

### 3.4. QM/MM Docking and Gold Results

#### 5tz1

**Figure 2:**
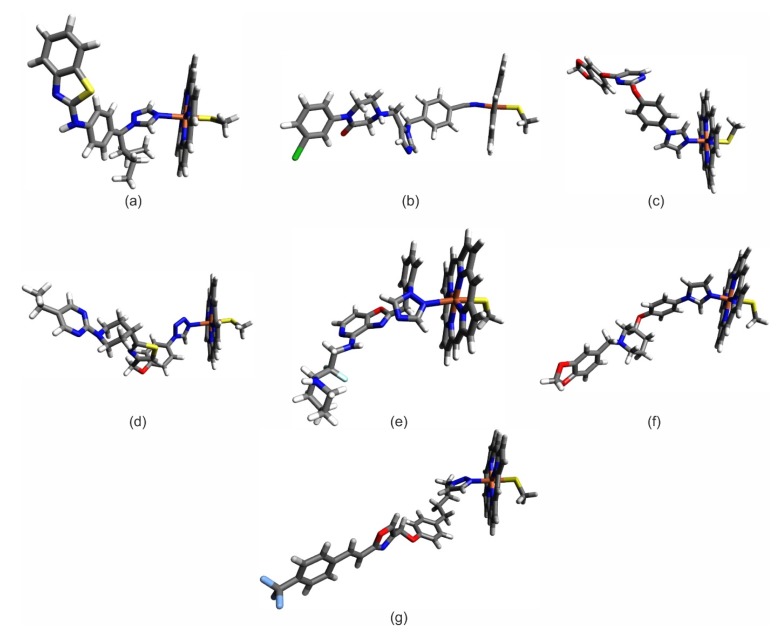
All seven finally selected lead compounds bound to the active center in 5tz1. Only the quantum mechanics (QM) region atoms are shown: (**a**) DB13083, (**b**) DB07227, (**c**) DB07008, (**d**) DB12345, (**e**) DB04591, (**f**) DB07011, and (**g**) DB12682.

Table 6 displays the QM/MM scoring energy for all the hit compounds that have been found to pass the Autodock 5a step for both approaches. For the evaluated six fungicides, the average score value is –73.93 kcal/mol and this value is the closest to voriconazole. Therefore, the QM/MM score of voriconazole was selected as the final QM/MM score criterion cut-off for the selection of lead compounds. When the QM/MM scores for fungicides are linearly matched with the literature inhibitory effect values (Table 6 numbers in brackets from Reference [11]), the obtained *R*^2^ value is 0.536. If the result for the water molecule is included in that linear correlation with 0% inhibition, then the *R*^2^ value is 0.713 (Supplementary Table S13). For comparison, in Reference [35] the linear *R*^2^ between the %Inhibition and Δ*G*_pred_ equals 0.607 on a different antifungal system (see Table 3 in Reference [35] and match the %Inhibition column with the Δ*G*_pred_ column). Therefore, we find our QM/MM calculations credible for estimating the binding affinity of the tested hit compounds. With the determined cut-off value for voriconazole, these (*) seven Drugbank compounds (Table 6) have passed the QM/MM step selection criteria (Scheme 1 and Scheme 2). Prior to that, they have also been verified by Gold docking. The GoldScores obtained for these seven hit compounds are all above the median GoldScores determined for the 70 known Cyp51 target fungicides (Supplementary Table S8). The analysis of 100 conformations obtained with the Gold docking program for the most selected compounds coincides with the Autodock4 geometry used in the input for the ORCA runs. In particular, for all seven compounds there is at least one conformation with the proximity of the nitrogen atom to the iron atom d(Fe-N) ≤ 3Å. Five out of seven compounds have several conformations with d(Fe-N) ≤ 3Å. This means that all seven hit compounds have passed all seven steps in filtering criteria and are finally selected as lead compounds for the 5tz1 CYP51 protein target: DB13083, DB07227, DB07008, DB04591, DB07011, DB12345, and DB12682. Table 7 shows a list of the interacting amino-acid residues of the 5tz1 target (within 4 Å) with any lead compound atom. These seven selected DB compounds obey the Lipinski rule of five and should be considered as potential novel inhibitors of the Cyp51 target. Figure 2 shows their binding to the heme cofactor.

Information about each selected lead compound is available from Drugbank [36], and we report the summarized data in Supplementary information note 3.

As can be seen from Figure 2, all the lead compounds have a nitrogen atom coordinating iron. Conformations with the oxygen atom bound to the iron atom were also considered, but the result has shown a weaker binding affinity. For that reason, the proximity and orientation of the nitrogen atoms were of delicate importance when deciding which conformation to use for the QM/MM optimizations. Of course, there are examples of hit compounds with a azole nitrogen coordinating iron, but they are below the energy cut-off (e.g., DB12017); that is why the full list of interacting amino-acid residues is depicted in Table 7 for all the lead compounds. The list of interacting amino-acids for the lead compounds seems to significantly coincide with oteseconazole (Table 7). In particular, both oteseconazole and all seven selected lead compounds interact with the following amino-acid residues: Tyr118, Leu121, Tyr132, Phe228, Pro230, Phe233, Gly307, Thr311, Leu376, His377, Phe380, and Met508. Additionally, for oteseconazole, when this list of interacting amino-acids obtained by our calculations (Table 7) was compared with the amino-acid list of the crystallographic data for oteseconazole (i.e., in Reference [11]), our calculations reproduced all the interacting amino-acids, with only the following excess interacting amino-acids: Gly65, Met306, Gly308, and Ser506.

### 3.5. 5hk1 Protein

The results of the ORCA DFT QM/MM gradient calculations for the charged and uncharged ligand systems are below (Table 8) and more can be found in Supplementary Table S14. Only the results of the same charged systems can be mutually compared. For the uncharged species, only DB07075, DB08622, and DB08746 obtained a higher (i.e., more negative) QM/MM score than dodemorph. For the charged species (+1), DB08622 and DB08746 obtained a higher QM/MM score than dodemorph, and since they also did as uncharged species, they passed the final QM/MM step. Although DB06555 did have a higher score than dodemorph in a charged state, it did not have it in an uncharged state, and we cannot be sure that it passed this QM/MM step. For the charged species (+2), DB07075 had a higher QM/MM score than the reference compound (at +2 charge), and since DB07075 has already had a higher QM/MM score than (other) reference compounds in an uncharged state, it passed the final QM/MM test. The Gold Chemscores for these three hit compounds are at or above the median, determined for the 21 known Erg2 target fungicides and pharmacologically correlated inhibitors of the sigma 1 receptor (Tables S9, S10), with DB07075 being fourth from the top value. This means that DB07075, DB08622, and DB08746 have passed all the seven filtering steps and are finally selected as Erg2 lead compounds. It is, however, worth noting that DB06555 obtained the highest Gold Chemscore and was very close to being chosen as a lead compound.

**Figure 3:**
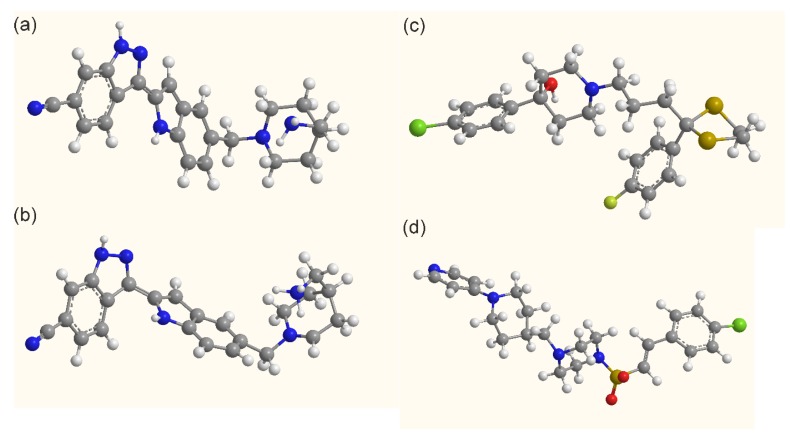
(**a**) DB07075 free ligand optimized in water solution (PCM). (**b**) DB07075 ligand optimized in the bound state with the 5hk1 protein. Please note that when compared with free ligand (**a**), in this case (**b**) the C2 piperidine atom is negative, lacking one proton which is translocated to the -NH_2_ group forming -NH_3_^+^. (**c**) The DB08622 ligand is optimized in the bound state with the 5hk1 protein (nothing unusual). (**d**) The DB08746 ligand is optimized in the bound state with the 5hk1 protein (nothing unusual). This might explain why the QM/MM score is much more negative for DB07075 than for DB08622 or DB08746. Proton translocation, when binding from free ligand to the complex, took place only with DB07075.

The reason why DB07075 in its uncharged state (Table 8) obtained the highest QM/MM score for binding energy might be attributed to a translocated proton in the minimized protein-ligand complex structure. The proton translocated from the piperidine C2 atom to the NH_2_ group forming the secondary carbon -CH^−^- and the -NH_3_^+^ group bound to the protein COO^–^ group obviously created some kind of negative-positive-negative bridge. That happened only to DB07075, not to any other compound (Figure 3).

Very recent literature has already published a detailed analysis of a 3D Quantitative Structure-Activity Relationship (QSAR) and docking study on the σ1 receptor using the same 5hk1 PDB [37], but neither the QM/MM docking score results were carried out there nor were any of these selected novel lead compounds presented there. The crystallographic structure PDB of the σ1 receptor is a hot novel PDB acquirable structure. Our docking study has shown that generally conformations with ligands very close to the Glu172 unit yield the strongest binding, which follows all previous literature notifications [37]. In addition, the selected highest QM/MM-scored compound DB07075 also yielded the highest docking score both in Autodock4 and in Autodock vina when compared to all the other hit and fungicide compounds. Information about each selected lead compound is available from Drugbank [36] in Supplementary information note 3.

## 4. Conclusions

We carried out a rigorous machine learning and docking-based drug repurposing study for the search of novel antifungal lead compounds from the Drugbank database. Firstly, we constructed two approaches with ensembles of classification models for the repurposing with a verified cross-validated and test-validated high degree of accuracy. The extracted set of compounds was further scrutinized, and only those having specific chemical functional groups were recognized to be potential Cyp51 inhibitors or Erg2 inhibitors. For Cyp51, we used the 5tz1 protein target, and for Erg2, we approximated the filtering with the homologically similar σ1 receptor target. Finally, we docked all hits to corresponding targets using Autodock4 and Autodock vina, and additionally verified the docking scores using the Gold molecular docking program. We carried out QM/MM gradient simulations for the best-docked positions of the top-scoring ligands. Our results suggest novel lead compounds that passed all seven filtering steps (Scheme 1 and Scheme 2): DB13083, DB07227, DB07008, DB04591, DB07011, DB12345, and DB12682 for the Cyp51 target (5tz1); and DB07075, DB08622, and DB08746 for the Erg2. Since none of these compounds, to the best of our knowledge, has any record to date regarding either antifungal activity or any record of the inhibition of the Cyp51 target of *Candida albicans* or similar targets (or even the σ1 receptor target), we find our contribution significant for future in vitro studies of these DB compounds or their derivatives.

## Figures and Tables

**Table 1:** Results of Uninformative Variable Elimination Partial-Least Squares UVE-PLS, UVE-PLS-RF, stepwise multilinear regression (FS-MLR), and UVE-PLS-FS-MLR for mode of action MOA classification and the number of selected compounds from Drugbank. The model accuracy by the percent of correct classification (PCC) of cross-validation ‘(CV)’ on the training set and the test set ‘te’.

No Class.	Method	Nvar	PCC(CV)	PCCte	Passed Step II (III)	No. Hit (Fung) *
11	UVE-PLS-DA	185	66.2	67.1	75 (35)	34 (13)
11	UVE-PLS-RF	185	65.0	76.8	62 (49)	34 (16)
4	UVE-PLS-DA	324	77.3	77.6	636 (2)	1 (0)
4	UVE-PLS-RF	324	76.1	82.4	412 (2)	2 (2)
3	UVE-PLS-DA	115	82.8	84.1	273 (24)	14 (5)
3	UVE-PLS-RF	115	81.6	86.6	334 (20)	17 (7)
11	UVE-PLS-FS-MLR	21	68.7	63.4	64 (34)	28 (10)
11	FS-MLR	30	71.8	65.9	24 (24)	10 (2)
4	UVE-PLS-FS-MLR	6	65.7	60.8	0 (0)	0 (0)
4	FS-MLR	14	76.9	66.4	106 (51)	24 (5)
3	UVE-PLS-FS-MLR	11	84.0	73.2	3 (3)	0 (0)
3	FS-MLR	20	91.4	79.3	100 (18)	8 (1)

* “No. hits” denotes the number of Drugbank hit compounds that passed step 4 (number in brackets denotes the already literature-established fungicides among these hits).

**Table 2:** Forward Stepwise Limited Correlation Random Forest (FS-LM-RF) results.

No Class.	Corr Gap	Nvar	PCC(CV)	PCCte	Passed Step II (III)	No. Hits (Fung) *
11	0.5	16	68.1	65.9	25 (24)	21 (7)
11	0.6	14	64.4	72.0	57 (44)	36 (12)
11	0.7	22	71.8	68.3	62 (40)	27 (8)
11	0.8	17	65.6	70.7	97 (22)	14 (4)
11	0.9	14	69.9	76.8	67 (35)	24 (11)
4	0.5	17	72.9	75.2	402 (11)	8 (4)
4	0.6	13	80.1	80.0	437 (13)	7 (6)
4	0.7	18	79.7	80.0	468 (11)	5 (3)
4	0.8	17	78.5	73.6	296 (26)	21 (13)
4	0.9	18	78.5	72.8	328 (10)	4 (3)
3	0.5	15	83.4	80.5	79 (28)	21 (6)
3	0.6	19	79.8	85.4	314 (46)	35 (13)
3	0.7	12	81.6	86.6	94 (18)	11 (8)
3	0.8	18	85.3	86.6	218 (2)	1 (0)
3	0.9	16	83.4	80.5	176 (13)	13 (5)

* “No. hits” denotes the number of Drugbank hit compounds that passed step 4 (number in brackets denotes the already literature-established fungicides among these hits).

**Table 3:** Results of the two-class regression. Columns 2–5 all denote the average PCC among a certain number of experiments. PCCTP(te) denotes the percent of correct classification of the true positives for the test set (details in Table S6b).

Method	PCC(CV)	PCC(te)	PCCTP(te)	PCC (27 Fung)	Comp. Sel. (Hits) *	Total No. Rep.
UVE-PLS	98.14	98.5	71.33	77.14	42	365
UVE-RF	96.93	98.54	70.0	91.85	26	148
RF-LM-FS	98.40	98.37	66.55	90.37	78	300
All	97.95	98.46	69.33	85.27	100 (37)	813

* Number in brackets is the total number of hit compounds selected with at least 3 selections. Other numbers in the sixth column denote the numbers of the selected compounds from Drugbank with only one selection.

**Table 4:** Analysis of the 100 docked conformations of the known fungicides (Δ*G* in kcal/mol) taking into account both the energy and geometry of the docked complex (see also Table S7).

	Δ*G*(100) ^(a)^	Δ*G*(<3Å) ^(b)^	α ^(c)^		Δ*G*(100) ^(a)^	Δ*G*(<3Å) ^(b)^	α ^(c)^
albaconazole	−9.78	−9.08	92.9	flutriafol	−7.89	−7.43	164.9
bifonazole	−10.02	−10.02	97.0	fosfluconazole	−10.82	−7.18	157.4
bitertanol	−9.57	−8.75	119.9	lanoconazole	−8.93	−8.52	106.7
bromuconazole	−8.99	−8.28	168.9	levoketoconazole	−12.88	−12.56	179.3
butoconazole	−10.14	−9.51	148.10	luliconazole	−8.97	−8.88	117.1
climbazol	−7.92	−7.54	160.4	mefentrifluconazole	−9.38	−8.58	115.2
clotrimazole	−9.87	−9.77	157.9	metconazole	−8.84	−8.83	169.8
croconazole	−9.28	−8.77	170.1	miconazole	−10.09	−9.94	167.0
eberconazole	−9.98	−9.98	142.6	myclobutanil	−8.7	−8.70	134.3 ^(d)^
econazole	−9.9	−9.90	169.7	neticonazole	−7.61	−7.28	166.0
efinaconazole	−8.15	−7.66	160.3	nuarimol	−8.58	−8.53	147.2
epoxiconazole	−9.4	−8.88	164.1	omoconazole	−10.18	−10.18	169.7
etaconazole	−8.51	−7.78	163.4	oxiconazole	−10.78	−10.78	168.8
fenarimol	−9.28	−8.98	154.3	oxpoconazole	−10.17	−9.99	174.4
fenbuconazole	−9.94	−9.83	159.5	pefurazoate	−7.81	−7.16	110.8
fenticonazole	−11.97	−11.97	164.7	penconazole	−7.76	−7.26	166.8
fluconazole	−6.76	−6.76	170.4	posaconazole	−13.81	−13.04	140.9
fluquinconazole	−8.98	−8.54	143.3	prochloraz	−8.14	−7.77	122.6
furconazole-cis	-8.15	−8.15	117.1	propiconazole	−8.64	−8.40	148.0
hexaconazole	−7.68	−6.98	174.6	prothioconazole	−8.12	−7.98	150.2 ^(e)^
imazalil	−7.73	−7.73	178.7	ravuconazole	−10.69	−9.97	171.9
imibenconazole	−9.66	−9.46	154.5	sertaconazole	−10.6	−10.6	129.0
ipconazole	−9.26	−8.92	176.9	sulconazole	−9.76	−9.76	162.4
ipfentrifluconazole	−9.53	−9.53	124.0	tebuconazole	−8.44	−7.30	145.9
isavuconazole	−11.14	−10.22	162.3	tetraconazole	−7.4	−6.53	170.6
isoconazole	−10.02	−9.30	147.4	tioconazole	−9.16	−9.16	134.4
itraconazole	−13.8	−12.52	146.1	triadimenol	−7.98	−7.36	138.7
ketoconazole	−12.59	−12.59	174.7	triticonazole	−9.64	−8.37	153.1
cyproconazole	−7.72	−7.72	166.6	voriconazole	−7.75	−7.75	170.0
difenoconazole	−11.09	−11.09	158.8	oteseconazole	−10.39	−10.24	152.4 ^(f)^
diniconazole−M	−8.7	−7.41	109.7	Average	−9.43	−9.01	150.8

(a) Minimum energy conformation of all the 100 conformations considered. (b) Represents the energy of the conformation that has the lowest binding energy (ΔG), but only in subsets among those conformations that contain an Fe-N distance of < 3Å. Others containing Fe-O < 3Å or Fe-Cl < 3Å are not mentioned in this table. (c) Displayed Fe-N-(R)C angle (in degrees, see Figure 1) representing the conformation that has the highest Fe-N-(R)C angle of coordinated ligand among those conformations containing an Fe-N < 3Å. The energy of such a conformation Δ*G*(max α) is given in Supplementary Table S7. The following rings are considered: imidazole, triazole, tetrazole, pyridine, pyrimidine. (d) It is about the Fe-N-C angle, where C denotes nitrile (C≡N) carbon and N denotes nitrile nitrogen. (e) Triazolithione ring. (f) The highest angle for oteseconazole is 174.7° but involving the Fe-coordinating N(26) tetrazole atom which is tetrazole N3, not tetrazole N4, and is therefore different then the specific coordinating N(27) atom in literature. The maximum angle for N(27) is 152.4, as displayed, and it was used in QM/MM.

**Table 5:** Analysis of step 5a using the 100 docked conformations of the hit compounds (Δ*G* in kcal/mol) for the 5tz1 hit compounds that passed prefiltering (so with Δ*G*(10) < −10.1 kcal/mol), taking into account both the energy (in kcal/mol) and geometry of the docked complex.

Hit Comp.	Comment, Default Fe-N<3Å Con-Taining, Otherwise as Commented	For QM/MM	Hit Comp.	Comment, Default Fe-N<3Å Con-Taining, Otherwise as Commented	For QM/MM
DB12623	No Fe-N, but with Fe-O, E −16.1	Yes	DB04107	No Fe-N, but with Fe-O, E −10.4	No
DB08387	max ∡Fe-N-®C of 99.5°	No	DB04600	2 × max ∡Fe®(R)C: 125°, 123°	Yes
DB00354	No dist(Fe-X) < 3Å (X=N or O)	No	DB07227	max ∡Fe-N-C of 146.7 + mode **	Yes × 2
DB08746	No Fe-N, but with Fe-O, *E* −10.8	No	DB06021	No dist(Fe-X) < 3Å (X=N or O)	No
DB12345	max ®-N-(R)C of 153.7°	Yes	DB12218	max ∡Fe-N-(R)C of 147.6°	Yes
DB08745	No Fe-N, but with Fe-O, E −16.1	Yes	DB02917	No Fe-N, but with Fe-O, E −10.6	No
DB07578	max ∡Fe-N-(R)C of 142.3°	Yes	DB12640	No dist(Fe-X) < 3.5Å (X = any!)	No
DB12561	max ∡Fe-N-(R)C of 119.8°	No	DB00699	No dist(Fe-X) < 3Å (X=N or O)	No
DB12017	Fe-O, *E* -11.16, and vina result *	Yes × 2	DB13113	max ∡Fe-N-(R)C of 141.4°	Yes
DB11679	No Fe-N, but with Fe-O, E −11.0	No	DB00737	No dist(Fe-X) < 3Å (X=N or O)	No
DB06834	No dist(Fe-X) < 3Å (X=N or O)	No	DB07255	No Fe-N, but with Fe-O, E −9.7	No
DB08922	No Fe-N, but with Fe-O, E −10.1	No	DB12364	No dist(Fe-X) < 3.5Å (X = any!)	No
DB04591	max ∡Fe-N-(R)C of 156.1°	Yes	DB01149	No dist(Fe-X) < 3Å (X=N or O)	No
DB12644	max ∡Fe-N-(R)C of 114.6°	No	DB07008	max ∡Fe-N-(R)C of 145.7°	Yes
DB04960	No dist(Fe-X) < 3Å (X=N or O)	No	DB02491	No Fe-N, but with Fe-O, E −10.2	No
DB13083	max ∡Fe-N-(R)C of 156.2°	Yes	DB09195	max ∡Fe-N-(R)C 137.1° (only)	No
DB12963	No dist(Fe-X) < 3.5Å (X = any!)	No	DB07011	max ∡Fe-N-(R)C of 149.3°	Yes
DB04957	No dist(Fe-X) < 3Å (X=N or O)	No	DB02706	No Fe-N, but with Fe-O, E −15.0	Yes
DB07878	No Fe-N, but with Fe-O, E −11.0	No	DB08560	No Fe-N, but with Fe-O, E −10.5	No
DB12682	max ∡Fe-N-(R)C of 153.8°	Yes			

* Two different binding groups of ligands oriented to Fe were selected, one with Adt4 regarding the COO group, and the other regarding the azole group but selected with Autodock vina, although maximum angle was only 139°. ** Two different binding groups of ligands oriented to Fe were selected, both with Adt4, one regarding the nitrile group with a max. ∡Fe-N-C of 146.7, and the other regarding imidazole N3, with two max. ∡Fe-N-C of 134° and 130°.

**Table 6:** QM/MM score results of the hit compounds (with Drugbank numbers), six fungicides (clotrimazole-voriconazole), and water (H_2_O) used for a reference comparison. Presented is Gibbs free binding energy with all (including ZPE correction and entropy) applied corrections. The orientation bound to the iron center with the lowest energy is presented. More detailed tables can be found in Supplementary Tables S11 and S12.

Compound	Δ*G*/kcal/mol	Compound	Δ*G*/kcal/mol
clotrimazole (fungicide)	−37.60 (78) ^(d)^	DB07008	−124.67 *
fluconazole (fungicide)	−46.37 (54) ^(d)^	DB12623	−71.59
miconazole (fungicide)	−91.74 (79) ^(d)^	DB07578	−70.49
ketoconazole (fungicide)	−86.58 (85) ^(d)^	DB12017 ^(b)^	−70.17
oteseconazole (fungicide)	−106.36 (98) ^(d)^	DB04591	−82.30 *
*voriconazole (fungicide)*	*−74.92* (84) ^(d)^	DB07011	−77.96 *
water molecule (H_2_O)	−12.22	DB08745	−57.15
DB13083	−82.73 *	DB13113	−31.15
DB04600	−225.38 ^(a)^	DB12345	−96.20 *
DB07227 ^(b)^	−128.79 *	DB12218	- ^(c)^
DB02706	−59.96	DB12682	−115.59 *

* Hit compounds that passed the QM/MM step, due to stronger binding than voriconazole and without a negative note. ^(a)^ Negative note: the compound decomposes due to unstable C-O-S(OH)2NH2 in the active center, which implies that it could decompose in similar conditions but with different proteins and in many different sites. ^(b)^ Presented is the stronger energy binding orientation; for DB07227, it is nitrile mode and for DB12017, azole mode. ^(c)^ Negative note: an attempt with QM/MM gradient minimization has been made and, due to the repulsion steric effect from the neighboring N-H group, the calculations ultimately failed to converge. ^(d)^ Number in brackets “( )” for the corresponding fungicide denotes the percent of CYP51 inhibition determined in Reference [11].

**Table 7:** List of interacting amino-acid residues with the selected docked fungicides and determined 5tz1 lead compounds. Each interaction is defined as the distance within 4.0 Å between any ligand atom and any protein amino-acid atom (so only heme atoms are excluded and therefore not taken into account).

Docked Comp.	No. Inter.	List of Interacting Amino-Acid Residues
voriconazole (fung)	283	Tyr118, Leu121, Thr122, Phe126, Ile131, Tyr132, Phe228, Gly303, Ile304, Gly307, Gly308, Thr311, Leu376, Ser378, Ile379, Met508
oteseconazole (fung)	383	Tyr64, Gly65, Tyr118, Leu121, Thr122, Phe126, Ile131, Tyr132, Phe228, Pro230, Phe233, Gly303, Ile304, Met306, Gly307, Thr311, Leu376, His377, Ser378, Phe380, Tyr505, Ser506, Ser507, Met508
DB13083 (lead)	406	Tyr118, Leu121, Thr122, Phe126, Ile131, Tyr132, Phe228, Pro230, Phe233, Gly303, Ile304, Gly307, Gly308, Thr311, Leu376, His377, Ser378, Ile379, Phe380, Ser507, Met508
DB07227 (lead)	354	Tyr64, Gly65, Leu87, Leu88, Tyr118, Leu121, Tyr132, Phe228, Pro230, Phe233, Gly307, His310, Thr311, Leu376, His377, Ser378, Ile379, Phe380, Tyr505, Ser506, Ser507, Met508, Val509
DB07008 (lead)	312	Tyr118, Leu121, Tyr132, Phe228, Pro230, Phe233, Gly307, Gly308, Thr311, Leu376, His377, Ser378, Ile379, Phe380, Tyr505, Ser506, Ser507, Met508, Val509
DB12345 (lead)	477	Tyr64, Gly65, Tyr118, Leu121, Thr122, Tyr132, Phe228, Pro230, Phe233, Gly307, Thr311, Leu376, His377, Ser378, Ile379, Phe380, Tyr505, Ser506, Ser507, Met508
DB04591 (lead)	442	Tyr64, Tyr118, Leu121, Thr122, Phe126, Ile131, Tyr132, Phe228, Pro230, Phe233, Gly303, Ile304, Gly307, Gly308, Thr311, Pro375, Leu376, His377, Ser378, Phe380, Tyr505, Ser507, Met508, Val509, Val510
DB07011 (lead)	401	Tyr64, Gly65, Pro68, Tyr118, Leu121, Tyr132, Phe228, Pro230, Phe233, Gly307, Gly308, His310, Thr311, Leu376, His377, Ser378, Ile379, Phe380, Tyr505, Ser506, Ser507, Met508, Val509
DB12682(lead)	377	Ala61, Tyr64, Gly65, Leu87, Leu88, Tyr118, Leu121, Thr122, Phe126, Ile131, Tyr132, Phe228, Pro230, Phe233, Gly303, Ile304, Gly307, Thr311, Leu376, His377, Ile379, Phe380, Tyr505, Ser506, Met508, Val509

**Table 8:** QM/MM docking results of the literature ligand compound “(l with reference)”, and the fungicide (dodemorph) and hit compounds (hit) analyses for both the uncharged species and for the ionized species at pH 7.4 (with charge). Presented is the Gibbs free binding energy with ZPE, BSSE, and dispersion correction applied (Δ*G*).

Uncharged Species	Δ*G*/kcal/mol	Compounds at pH 7,4 (Charged)	Δ*G*/kcal/mol
Pentazocine (l) [20]	+141.41	dodemorph (fung) (+1)	−294.39 (r)
dodemorph (fung)	−54.39 (r)	tamoxifene (l) (+1)	−285.95
opipramol (l) [20]	−55.16	Pentazocine (l) (+1)	−102.98
PD144418 (l) [37]	−86.53	PD144418 (l) (+1)	−367.39
tamoxifene (l) [20]	−45.94	DB06555 (hit) (+1)	−295.03
DB07075 (hit)	−185.13*	DB08622 (hit) (+1)	−319.22 *
DB07075 (hit) ^(a)^	−200.68	DB08746 (hit) (+1)	−370.02 *
DB08622 (hit)	−65.06 *	DB02491 (hit) (+1)	−159.12
DB08746 (hit)	−55.11 *	DB00637 (hit) (+1)	−163.40
DB12345 (hit)	−25.24		
DB00637 (hit)	+16.96		
DB06555 (hit)	−50.70	opipramol (l) (+2)	−498.73 (r)
DB02491 (hit)	−8.19	DB07075 (+2)	−519.81 *

* Hit compounds that passed the QM/MM step due to stronger binding both as uncharged species relative to dodemorph (r) and in charged state at a pH of 7.4 relative to dodemorph ((r) as +1 species) or opipramol ((r) as +2 species). ^(a)^ Same hit (DB07075) as in the row above but with a repeated run to verify the calculated QM/MM score again, with the ORCA input geometry used from a slightly different Autodock4 output conformation.

## Supplementary Materials

Including 14 Supplementary Tables (S1–S14, with S3a-S3b and S5A-S5C, S6A-S6B) in the spreadsheet and word tables, with possible notes and comments near them; one Supporting Instructions Note; one Supplementary Comment; one Supplementary List; and three Supporting Information Notes (which might include Supporting Table(s)). The Drugbank open structures sdf file was downloaded on 02-04-2019.
