# Supplementary material for: Combined Machine Learning and Molecular Modelling Workflow for the Recognition of Potentially Novel Fungicides
Source: Molecules. 2020 May 8;25(9):2198. doi: 10.3390/molecules25092198 (PMC7249108; doi:10.3390/molecules25092198)
Supplement: Supplementary file 1 [file molecules-25-02198-s001.zip › Revised_Supplementary/Supplementary_comment_1.docx]

Drugbank compounds with C atom count>40 were excluded. The reasons why are the following. Molecules with 40 C (or more) have (almost surely) molecular weight > 500 Da and that usually implies a possible collision with Lipinski rule of five. Too large molecules, have a small chance of arriving to the target's active site, especially in the appropriate way, so we decided to exclude those molecules. In addition, even the molecules as ketoconazole and itraconazole have molecular weight larger than 500 Da, but smaller number of C atoms than 40. Itraconazole is the heaviest class G fungicide (705.6 g/mol) but it would still be included in selection since its C atom count is 35. We did not want to include even larger molecules than itraconazole for possible novel hit compounds of class G, as there is no class G fungicide today with n(C)>40. Fungicide of the highest C-atom count is posaconazole of n(C)=37 (with molecular weight of 700.8 g/mol). Otherwise novel hit compounds might also not be within descriptor limits defined by our class G fungicide dataset, as is required by the first selection criterion (Scheme 1). And many descriptors describe either directly or indirectly the molecular size.
