# Supplementary material for: Combined Machine Learning and Molecular Modelling Workflow for the Recognition of Potentially Novel Fungicides
Source: Molecules. 2020 May 8;25(9):2198. doi: 10.3390/molecules25092198 (PMC7249108; doi:10.3390/molecules25092198)
Supplement: Supplementary file 1 [file molecules-25-02198-s001.zip › Revised_Supplementary/Supplementary_list_1.docx]

List of all calculated molecular descriptor groups:

AcidicGroupCount, ALOGP, APol, AromaticAtomsCount, AromaticBondsCount, AtomCount, Autocorrelation, BaryszMatrix, BasicGroupCount, BCUT, BurdenModifiedEigenvalues, CarbonTypes, ChiChain, ChiCluster, ChiPathCluster, ChiPath, Constitutional, Crippen, DeTourMatrix, EccentricConnectivityIndex, EStateAtomType, ExtendedTopochemicalAtom, FMF, FragmentComplexity, HBondAcceptorCount, HBondDonorCount, HybridizationRatio, InformationContent, KappaShapeIndices, LargestChain, LargestPiSystem, LongestAliphaticChain, MannholdLogP, McGowanVolume, MDE, MLFER, PathCount, PetitjeanNumber, RingCount, RotatableBondsCount, RuleOfFive, Topological, TopologicalCharge, TopologicalDistanceMatrix, TPSA, VABC, VAdjMa, WalkCount, Weight, WeightedPath, WienerNumbers, XLogP, ZagrebIndex
