# Supplementary material for: Combined Machine Learning and Molecular Modelling Workflow for the Recognition of Potentially Novel Fungicides
Source: Molecules. 2020 May 8;25(9):2198. doi: 10.3390/molecules25092198 (PMC7249108; doi:10.3390/molecules25092198)
Supplement: Supplementary file 1 [file molecules-25-02198-s001.zip › Revised_Supplementary/Supplementary_table_S5A-B.docx]

Table S5A. FS-LM-RF model descriptors.

| no. class  cor. | Selected PADEL molecular descriptors for each classification model |
| --- | --- |
| 11, 0.5 | SHBd, MDEC-13, SdssC, BCUTw-1l, ETA_Shape_Y, minsssN, MDEC-24, WTPT-5, AATSC1i, ATSC2m, MATS3s, minHBd, VCH-7, GATS2m, MATS4v, AATSC6c" |
| 11, 0.6 | MDEC-24, AATSC5p, BCUTw-1l, MATS4c, MATS3m, MATS1c, MDEC-13, JGI5, minsCl, minHBd, GATS1s, minsssN, ATSC3s, MDEN-23 |
| 11, 0.7 | SCH-5, mindssC, minaaN, maxHBd, SdssC, MDEC-24, minwHBa, AATS5s, MDEC-13, SsssN, BCUTw-1l, maxHCsats, MDEC-22, ATSC3s, XLogP, WTPT-5, MDEC-33, minHother, MATS1c, minHBa, MATS1i, GATS5c |
| 11, 0.8 | maxHCsats, BCUTp-1l, maxaasN, ETA_Shape_Y, AATS4m, ATSC1i, AATSC6c, VE1_Dze, SpMin7_Bhs, ASP-7, MDEC-11, piPC6, MATS1m, AATSC3m, CrippenLogP, MDEC-24, ATSC6m |
| 11, 0.9 | SHBd, mindssC, SCH-5, minaaN, MDEC-13, MDEN-23, nAtomP, AATS5s, MATS2m, minaasN, SdssC, AATSC6i, R_TpiPCTPC, MDEC-22" |
| 4, 0.5 | SCH-5, BCUTw-1l, AATSC2m, minHBa, SdsN, MATS1c, ATSC3m, AATSC5m, MATS3c, MATS1i, SsCl, ATSC4c, EE_Dzs, MDEC-33, ATSC2s, AATSC4eMDEC-13 |
| 4, 0.6 | SCH-5, MDEC-22, MDEN-23, XLogP, BCUTw-1l, AATSC2m, minHBa, MDEC-24, nBase, GATS3s, SdsN, MATS1c, maxHCsats |
| 4, 0.7 | SaasN, MDEC-22, ALogP, MDEN-23, VCH-5, XLogP, BCUTw-1l, AATSC2m, minHBa, MDEC-24, nBase, GATS3s, nHCsats, AVP-0, ALogp2, SdsN, MATS1c, maxHCsats |
| 4, 0.8 | SaasN, SaaN, MDEC-22, ALogP, MDEN-23, VCH-5, maxHother, maxHaaCH, SpMAD_Dt, XLogP, BCUTw-1l, nssCH2, AATSC2m, minHBa, MDEC-24, nBase, nRotBt |
| 4, 0.9 | SaasN, SCH-5, minaaN, SaaN, MDEC-22, ALogP, MDEN-23, VCH-5, maxHother, maxHaaCH, SpMAD_Dt, XLogP, BCUTw-1l, MATS5m, nssCH2, AATSC2m, SpMax3_Bhp, minHBa |
| 3, 0.5 | AATSC3s, GATS2m, AATSC8i, ATSC3m, minsssCH, MATS4e, ATSC1p, ATSC6i, AATSC4i, MATS1m, maxsssCH, VE3_DzZ, GATS8c, SHsOH, MDEC-24 |
| 3, 0.6 | SCH-5, MDEC-13, MATS3m, ATSC7i, GATS2e, MDEC-33, MATS1e, SsssN, maxHCsats, minHBd, GATS8v, ATSC6s , mindssC, ATSC6p, maxdsN, AATS5p, MATS3p, maxdO, ATSC8s |
| 3, 0.7 | SCH-5, ETA_Shape_Y, MATS3s, minsssN, MATS1e, MATS1c, nAtomP, GATS2c, maxHBd, AATSC3s, mindssC, MDEC-22 |
| 3, 0.8 | SaasN, MDEC-22, LipoaffinityIndex, SHBd, MDEC-24, MDEC-13, ETA_Shape_Y, mindssC, nAtomP, VCH-5, SpMax3_Bhi, ATSC4v, MDEN-23, MATS1e, SdssC, maxHCsats, ATSC5p, AATSC5m |
| 3, 0.9 | SCH-5, MDEN-23, mindssC, naasN, maxHother, maxaaN, MDEC-22, SpMax3_Bhv, SdssC, maxHaaCH, LipoaffinityIndex, SHBd, MATS3m, WTPT-5, maxHBd, GATS3s |

Table S5B. MLR model descriptors

| no. class, orig.var: raw/UVE-PLS | Selected PADEL molecular descriptors for each classification model |
| --- | --- |
| 11, raw | GATS3s, SaasN, nAtomP, AATS2m, VPC-4, SpMax4_Bhm, ATSC5s, GATS1s, ATS0s, SM1_Dzs, ATS2e, MATS6v, BCUTp-1l, ATS7e, SpMax_Dzs, SCH-7, SsssN, ATS8i, SRW6, SdssC, TIC5" |
| 11, UVE-PLS | GATS3s, SaasN, nAtomP, AATS2m, VPC-4, SpMax4_BhmATSC5s, GATS1s, ATS0s, SM1_Dzs, ATS2e, MATS6v, BCUTp-1l, ATS7e, SpMax_Dzs, SCH-7, SsssN, ATS8i, SRW6, SdssC, TIC5, |
| 4, raw | SaasN, nssCH2, nHother, AATS5p, ndsN, SpMin6_Bhv, ATSC8i, ATSC0c, TIC2, ATSC8v, MATS4s, SsOm, MATS6p, nX |
| 4, UVE-PLS | AATSC2i, ATS2m, ATS1e, AATSC0c, ATS8v, AATSC0v |
| 3, raw | naasN, nBondsD, ATSC8v, SwHBd, SssCH2, nsOm, nsssN, MATS1c, AATSC2i, ATSC5c, AATSC8v, SpMin3_Bhm, nHBint2, SC-4, AATS8p, MDEN-13, ALogP, AATS8s, MDEC-24, minddssS |
| 3, UVE-PLS | AATS3m, nH, ATS0e, ATS7s, ATS1p, AATS5v, ATS6v, ATS4e, ATS0m, nBr, nF |
