# Supplementary material for: Combined Machine Learning and Molecular Modelling Workflow for the Recognition of Potentially Novel Fungicides
Source: Molecules. 2020 May 8;25(9):2198. doi: 10.3390/molecules25092198 (PMC7249108; doi:10.3390/molecules25092198)
Supplement: Supplementary file 1 [file molecules-25-02198-s001.zip › Revised_Supplementary/Supporting_Information_Note_1.docx]

5tz1 protein contains protoporphyrin with hexacoordinated Fe, for which program Autodock4 defaultly assigns zero charge to Fe. In study Chaskar et al. [21] the authors considered Gasteiger charges +0 and +2 to Fe, and success rate for Fe +0 state was 34.4% for binding set and 32.5% for nonbinding set, on the other hand for Fe assigned +2 state success rate was 53.1% for binding set and 27.5 % [21]; which in our opinion means that state +2 performs better, so we assigned +2 charge to Fe. In addition, we also considered +0 charge for 8 literature the most known ligands considered, and these are: VT1161 (which has its complex pdb from rcsb database), ketoconazole, posaconazole, itraconazole, fluconazole, miconazole, voriconazole and clotrimazole [11]. We tried to establish a bond between Fe and each of eight mentioned ligands' nitrogen atom of imidazole/triazole/tetrazole ring using Autodock4 (with definition of a bond: distance(Fe-N) < 3Å). Only the model with +2.0 charge assigned to Fe established such bond for all 8 ligands. Finally, Gasteiger charge of +2.0 charge on Fe was used for all other cyp51 fungicides and for all obtained hits.

It should be noted that there is a difference between correct point charge and a formal charge. Formal charge used in this study is +3. But correct point charge (i.e. partial charge) used in simulations depends on environment, level of theory and the size of QM system. Due to electron delocalization over the whole analyzed system, the partial charge on iron will always be lower than formal charge. Thus, electron and charge delocalization minimizes the atomic charges The only question is how much lower? In reference [Ref. 21 in article] the authors say that in In the employed CHARMM22 force field,[Ref. 1 below] the heme iron carries a charge of +0.24 (although they treat iron in formal charge of +2).

The additional reason why we used +2 charge for iron in Autodock4 is the fact that coordinating nitrogen atoms surrounding the iron should have the overall formal charge of -2, but their overall Gasteiger charge obtained by Autodock was roughly -1. To approximately compensate the difference of formal and Gasteiger charge of -1 (-2 - (-1) = -1), we had to lower iron charge, at least from +3 to +2. Of course that we tried to play by changing the Gasteiger charges of both iron and coordinated nitrogens, and later to play by modifying the Gasteiger charges of the whole Heme molecule according to literature [Ref. 2 below]. But, the results obtained either by our own modifications of Gasteigers charges (e.g. of Fe +3) or by using the charges of Ref. 2 (below) worsened the bonding between iron and literature fungicides (when compared to charge +2 on iron).

[1] Mackerell, A. D.; Bashford, D.; Bellott, M.; Dunbrack, R. L.; Evanseck, J. D.; Field, M. J.; Fischer, S.; Gao, J.; Guo, H.; Ha, S.; Joseph-McCarthy, D.; Kuchnir, L.; Kuczera, K.; Lau, F. T.; Mattos, C.; Michnick, S.; Ngo, T.; Nguyen, D. T.; Prodhom, B.; Reiher, W. E.; Roux, B.; Schlenkrich, M.; Smith, J. C.; Stote, R.; Straub, J.; Watanabe, M.; Wiórkiewicz-Kuczera, J.; Yin, D.; Karplus, M. All-Atom Empirical Potential for Molecular Modeling and Dynamics Studies of Proteins. J. Phys. Chem. B 1998, 102, 3586−3616.

[2] Angelo Danilo Favia, Andrea Cavalli,* Matteo Masetti, Angelo Carotti, Maurizio Recanatini. Three-Dimensional Model of the Human Aromatase Enzyme and Density Functional Parameterization of the Iron-Containing Protoporphyrin IX for a Molecular Dynamics Study of Heme-Cysteinato Cytochromes PROTEINS: Structure, Function, and Bioinformatics 62:1074–1087 (2006).
