# Supplementary material for: Combined Machine Learning and Molecular Modelling Workflow for the Recognition of Potentially Novel Fungicides
Source: Molecules. 2020 May 8;25(9):2198. doi: 10.3390/molecules25092198 (PMC7249108; doi:10.3390/molecules25092198)
Supplement: Supplementary file 1 [file molecules-25-02198-s001.zip › Revised_Supplementary/Supporting_information_note_2_and_Supplementary_table_S11.docx]

For 5tz1, the fact is that vast majority of literature important fungicides are imidazole and triazole compounds which at pH 7.4 are neutral uncharged species. Firstly, we conducted model calculation on non-hit and non-fungicide compound DB08192 which has both –COOH and –NH2 groups, to obtain more insight into 5tz1 ionization state docking. The results on the model compound have shown that in the protein active site, neutral uncharged species is more stable (QM/MM score −54.98 kcal/mol) than zwitterionic neutral species counterpart that exists at pH 7.4 (QM/MM score −33.08 kcal/mol). This leads to conclusion that using charged species (with possible addition of counter ions) is, at least, not expected to meaningfully improve binding score. And for that reason, defaultly all hit compounds were QM/MM docked neutral and uncharged. But for completeness of whole study, and despite computational costs we still considered charged ionization states for few compounds with po-sitive piperidine group at pH 7.4, and such results (with charged species) did not significantly change our final docking scores and conclusions regarding selection or non-selection of lead compounds. Independently of different ionization states, results in Autodock4 yield more solutions (i.e. modes of binding) for few compounds involving e.g. one conformation with oxygen atom bound to Fe and e.g. other conformation with nitrogen atom bound to Fe, or with more N atoms in molecule possible to bind to Fe. All these different modes of binding were also taken into account for QM/MM studies as separate parallel geometry optimization runs and other runs (single point, frequency runs, etc. as mentioned in the main article text).

Regarding different ionic states (positively charged species at PH = 7.4) of the same hit compounds, actually such are ONLY the following three compounds: DB07227, DB04591, DB07011. All these three mentioned hit compounds, in their uncharged states, have in term of absolute values higher QM/MM score than voriconazole (< −74.92), i.e. DB07227 (−128.79), DB04591 (−82.30), DB07011 (−77.96). We emphasize again higher QM/MM score is already obtained for these uncharged species. Therefore, calculations of their charged species (at pH 7.4) cannot alter any conclusion regarding their lead selection, since condition for passing voriconazole has already been met for all these three compounds. For this reason we did not want to put these results for charged species in the main article in order not to confuse the readership with more results for the same hit compounds. The same comment regards different orientations for two hit compounds: DB12017 and DB07227. We only put results for highest scored (in absolute terms, of course), which is for DB07227 nitrile orientation (or: C≡N-Fe) and for DB12017 azole orientation. So, these additional results are of secondary importance (even "borderline redundant importance"). So, we did consider different species and different orientations but in the main article we only show non-redundant information for each hit compound.

Under such completely non-obligatory circumstances, we even addionally show, for free, how we tried to make energies obtained for charged species comparable with uncharged species (HOMO-LUMO complex to ligand correction), see Supplementary table S12 with all comments and conclusions there.

**Table S11**. QM/MM docking results of model compound ("Mod DB08192"), six fungicides (clotrimazole-voriconazole), water (H_2_O) and hit compounds (hit), some of them in different ionization states ("-[ ]"), default is neutral, uncharged state; and some in different orientations when bound to iron center ("or:"). Presented is Gibbs free binding energy with all (including ZPE, entropy) applied corrections (other energies are in supp. table S12)

| Compound | Δ*G* / kcal/mol |  | Compound | Δ*G* / kcal/mol |
| --- | --- | --- | --- | --- |
| Mod DB08192 | −54.98 |  | DB07227 (hit) or: azoleN-Fe | −54.00 |
| Mod DB08192^–^ [-COO^−^] | −29.82 (−44.28) ^(b)^ |  | DB07227^+^ (hit) or: C≡N-Fe | −212.31 (−165.17)^(b)^ |
| Mod DB08192^+^ [-NH^+^] | −78.02 (−27.83) ^(b)^ |  | [-NH^+^] |  |
| Mod DB08192 [zwitter ion] | −33.08 |  | DB07008 (hit) | −124.67* |
| clotrimazole (fungicide) | −37.60 [78] |  | DB12623 (hit) | −71.59 |
| fluconazole (fungicide) | −46.37 [54] |  | DB07578 (hit) | −70.49 |
| miconazole (fungicide) | −91.74 [79] |  | DB12017 (hit) or: C=O-Fe | −47.42 |
| ketoconazole (fungicide) | −86.58 [85] |  | DB12017 (hit) or: azoleN-Fe | −70.17 |
| oteseconazole (fungicide) | −106.36 [98] |  | DB04591 (hit) | −82.30* |
| *voriconazole (fungicide)* | *−74.92* [84] |  | DB04591^+^ (hit) [-NH^+^] | −155.20 (−99.82) ^(b)^ |
| water molecule (H_2_O) | −12.22 |  | DB07011 (hit) | −77.96* |
| DB13083 (hit) | −82.73* |  | DB07011^+^ (hit) [-NH^+^] | −127.24 (−60.11) ^(b)^ |
| DB04600 (hit) | −225.38 ^(a)^ |  | DB08745 (hit) | −57.15 |
| DB07227 (hit) or: C≡N-Fe | −128.79* |  | DB13113 (hit) | −31.15 |
| DB12218 | - ^(c)^ |  | DB12345 (hit) | −96.20* |
| DB02706 | −59.96 |  | DB12682 | −115.59* |

* Finally recommended compound due to stronger binding than voriconazole and without any negative note.

^(a)^ Negative note: The compound decomposes due to unstable C-O-S(OH)2NH2 in the active center which implies that it could decompose in similar conditions but different proteins and in many different sites.

(b) Number in these brackets '( )' denotes binding energy after HOMO/LUMO(comp,lig) correction to make values obtained for ions, at least, roughly comparable with values obtained for neutral species see Suppl. table S12 and all explanations below.

^(c)^ Negative note: Attempt with QM/MM gradient minimization has been made and due to repulsion steric effect from neighbor N-H group, calculations ultimately failed to converge.

^(d)^ Number in brackets '[ ]' for corresponding fungicide denotes percent of CYP51 inhibition determined in Reference [11].
