# Supplementary material for: Combined Machine Learning and Molecular Modelling Workflow for the Recognition of Potentially Novel Fungicides
Source: Molecules. 2020 May 8;25(9):2198. doi: 10.3390/molecules25092198 (PMC7249108; doi:10.3390/molecules25092198)
Supplement: Supplementary file 1 [file molecules-25-02198-s001.zip › Revised_Supplementary/Supporting_information_note_3.docx]

DB13083 (377.17 Da, MLogP = 3.11 (Padel predicted), H-Bond Donors: 1 H-Bond Acceptors: 5) Talarozole. Citation from Drugbank: Talarozole has been investigated for the treatment of Psoriasis and Cutaneous Inflammation. It falls under cathegory Cytochrome P-450 Enzyme Inhibitors. Has a target in humans Cytochrome P450 26A1 (target CYP26A1 plas a role in retinoic acid metabolism). No records of antifungal activity.

DB07227 (405.14 Da, MLogP = 3.11, H Bond Donors: 0, Bond Acceptors: 4) 4-[(5-{[4-(3-Chlorophenyl)-3-oxopiperazin-1-YL]methyl}-1H-imidazol-1-YL)methyl]benzonitrile. Not available description on Drugbank and not avaialble categories. Targets in humans (gene names): FNTA, FNTB and PGGT1B (zinc ion binding). Indirect biomolecular record on pubchem [Ref. 1, below]: Cryptococcus neoformans protein farnesyltransferase (rcsb: 3Q7A). Has ADMET predicted features regarding CYP 450 targets with high probabilities.

DB07008 (374.10 Da, MLogP: 2.78, HBond Donors: 0 HBond Acceptors: 5). 4-(1,3-Benzodioxol-5-Yloxy)-2-[4-(1h-Imidazol-1-Yl)phenoxy]pyrimidine. Description and categories not available. Targets in humans: NOS2. Has ADMET predicted features regarding with more CYP 450 targets and with high probabilities.

DB04591 (439.19 Da, MLogP: 2.78, H-Bond Donors: 6 NBond Acceptors: 2). N-[2,2-Difluoro-2-[(2R)-piperidin-2-yl]ethyl]-2-[[2-(1,2,4-triazol-1-yl)phenyl]methyl]-[1,3]oxazolo[4,5-c]pyridin-4-amine. Description and categories not available. Targets in humans: F2 (Prothrombin). Has ADMET predicted features regarding CYP 450 targets with high probabilities.

DB07011 (377.17 Da, MLogP: 3.22. H-Bond Donors: H-Bond Acceptors: 5) (3s)-1-(1,3-Benzodioxol-5-Ylmethyl)-3-[4-(1h-Imidazol-1-Yl)phenoxy]piperidine. Description and categories not available. Targets in humans: NOS2. Has ADMET predicted features regarding CYP 450 targets with high probabilities.

DB12345 (448.18 Da, MLogP: 2.78, H-Bond Donors: 0 H-Bond Acceptors: 8). MBX-2982. MBX-2982 has been used in trials studying the treatment of Diabetes. Category: Sulfur compounds. No predicted ADMET properties avalable. Additional note on Drugbank: "*This drug is a stub and has not been fully annotated. It is scheduled to be annotated soon*" [36].

DB12682 (468.48 Da, MLogP = 3.11 (Padel predicted), H-Bond Donors: 0 H-Bond Acceptors: 4). Mubritinib. Mubritinib has been used in trials studying the treatment of Lung Neoplasm, Renal Neoplasm, Breast Neoplasm, Ovarian Neoplasm, and Pancreatic Neoplasm. No predicted ADMET properties avalable. Additional note on Drugbank: "*This drug is a stub and has not been fully annotated. It is scheduled to be annotated soon*" [36].

DB07075 (387.48 Da, MLogP = 3.33 (from Padel), H-Donor Count: 3, H-Acceptor Count: 4) 3-(5-{[4-(aminomethyl)piperidin-1-yl]methyl}-1H-indol-2-yl)-1H-indazole-6-carbonitrile. Description and categories not available. Targets in humans: CHEK1. Has many ADMET predicted features targets and with high probabilities. No records about any fungal activity or σ1 receptor activity.

DB08622 (452.05 Da, MLOgP = 3.33, H-Donor Count: 1, H-Acceptor Count: 2) 4-(4-chloro-phenyl)-1-{3-[2-(4-fluoro-phenyl)-[1,3]dithiolan-2-yl]-propyl}-piperidin-4-ol. Description and categories not available. Targets in humans: gag-pol. Has many ADMET predicted features targets and with high probabilities. No records about any fungal activity or σ1 receptor activity.

DB08746 (461.02 Da, MLogP = 3.11, H-Donor Count: 0, H-Acceptor Count: 5) 1-[[(1E)-2-(4-chlorophenyl)ethenyl]sulfonyl]-4-[[1-(4-pyridinyl)-4-piperidinyl]methyl]piperazine. Targets in humans: F10. No records about any fungal activity or σ1 receptor activity.

[1] <https://pubchem.ncbi.nlm.nih.gov/compound/216454#section=Biomolecular-Interactions-and-Pathways> accessed on 10/1/2020.
