# Supplementary material for: Combined Machine Learning and Molecular Modelling Workflow for the Recognition of Potentially Novel Fungicides
Source: Molecules. 2020 May 8;25(9):2198. doi: 10.3390/molecules25092198 (PMC7249108; doi:10.3390/molecules25092198)
Supplement: Supplementary file 1 [file molecules-25-02198-s001.zip › Revised_Supplementary/Supproting_Instructions_Note_1.docx]

10 steps for obtaining descriptor dataset of 8172 Drugbank compounds:

Step 1. On 9th April 2019 were downloaded all openstructes from Drugbank database. - drugbank_all_open_structures.sdf.zip

Step 2. Extract drugbank_all_open_structures.sdf.zip to open structures.sdf

Step 3. Load open structures.sdf with (already installed) OpenBabelGUI program and convert all structers to smiles using the option "Remove all but the largest contiguous fragment.

**One will obtaine (only) 9680 smiles** (i.e.9680 lines of smiles) in file 'open_structures.smiles'

Step 4. Now, use only smiles void of any numbers after it.

**Open python**

And use the following code:

#START of PROGRAM

import numpy as np

import pandas

import pubchempy as pcp

from pubchempy import get_compounds, Compound

num_lines=sum(1 for line in open('open_structres.smiles'))

s = ""

for i in range(num_lines):

with open('open_structres.smiles') as f:

line=f.read().split('\n')[i]

count=line.count('C')+line.count('c')

if ('Hg' not in line and 'Al' not in line and 'Au' not in line and 'Gd' not in line and '[Cl' not in line and 'Cu' not in line and '[Ag' not in line and 'Ga' not in line and 'Os' not in line and 'Ru' not in line and 'Zn' not in line and 'Re' not in line and 'Be' not in line and 'Mg' not in line and 'Ca' not in line and 'Fe' not in line and 'Co' not in line and 'Ni' not in line and 'Li' not in line and 'Na' not in line and 'K' not in line and 'Rb' not in line and 'Sr' not in line and 'Mn' not in line and 'Cr' not in line and 'Ge' not in line and 'Pd' not in line and 'Pt' not in line and 'Y' not in line and 'In' not in line and 'Tc' not in line and 'Ra' not in line and 'Sc' not in line and 'Mo' not in line and 'Ir' not in line and 'W' not in line and 'Ta' not in line and 'U' not in line and 'Zr' not in line and 'Cd' not in line and 'Rh' not in line and 'Pb' not in line and 'Sn' not in line and 'As' not in line and 'Sb' not in line and 'Tl' not in line and 'Bi' not in line and 'Po' not in line and 'At' not in line and 'Te' not in line and 'La' not in line and 'Ce' not in line and 'V' not in line and 'Ti' not in line and count<41):

s+=line+'\n'

else:

s+='\n'

f1=open('SMILES_corrected.txt','w+')

f1.write(s)

f1.close()

#END OF PROGRAM

You have just obtained in this step smiles for all compounds void of metals and void of compounds larger than n(c)>40 in 'SMILES_corrected.txt'

step 5. Open R with the following code:

#START of PROGRAM

library("ChemmineOB")

library("ChemmineR")

fileName="C:\\Users\\ojovic\\Desktop\\SMILES_corrected.txt"

con=file(fileName,open="r")

line=readLines(con)

sdfStr<-array(length(line))

for (i in 1:length(line)) {

sdfStr[i] = convertFormat("SMI","SDF",line[i], options=data.frame(names="gen3D",args=""))

if (i==1) {

write(sdfStr[i], "C:\\Users\\ojovic\\Desktop\\**sdf_2.txt**", sep = "\n")}

else {

write(sdfStr[i], "C:\\Users\\ojovic\\Desktop\\**sdf_2.txt**", sep = "\n",append=TRUE)}

}

t<-grep('OpenBabel',readLines("C:\\Users\\ojovic\\Desktop\\**sdf_2.txt**"))

t<-t-1

t<-t[-1]

con <- file("C:\\Users\\ojovic\\Desktop\\**sdf_2.txt**", open = 'r')

line <- readLines(con)

lime<-line[-t]

write(lime, "C:\\Users\\ojovic\\Desktop\\**sdf_2_output**.txt")

#END OF PROGRAM

You have just obtained text file in sdf format for all 9680 structures except those containing metals and those larger than n(c)>40, that is PERFECTLY FIT FOR padel program! (initial sdf file (open structures.sdf) was impossible to run in padel)

step 6. Create folder only for file **sdf_2_output**.txt and put it into that folder.

Step 7. Open Padel and calculate all 1D and 2D descriptors for that folder.

Step 8. Open R

#START of PROGRAM

library("ChemmineOB")

library("ChemmineR")

fileName2="C:\\Users\\ojovic\\Downloads\\open structures.sdf" *#Load the open structures.sdf in appropriate folder where that file is.*

con=file(fileName2,open="r")

line=readLines(con)

brojac<-0

t<-c()

for (i in 1:length(line)) {

if (line[i]=="> <DRUGBANK_ID>") {

brojac<-brojac+1

if (brojac==1) {

t<-i}

else {

t<-cbind(t,i)}

}}

t<-t+1

v<-c()

for (i in 1:length(t)) {

if (i==1) {

v<-line[t[i]]}

else {

v<-cbind(v,line[t[i]])}

}

write(t(v), "C:\\Users\\ojovic\\Downloads\\sdf_DB_numbers_test.txt")

#END OF PROGRAM

The result on 9th April was file with 9680 Drugbank ID-s in sdf_DB_numbers_test.txt

Step 9. Open both csv file created from padel program and sdf_DB_numbers_test.txt and copy a single column of sdf_DB_numbers_test.txt to output file of padel program – merge it.

Step 10. Now, delete all lines with any missing value. You will obtain exactly 8172 lines, each line with DB ID and 1444 calculated 1D and 2D descriptors.
